# Supplementary material for: On the fluorescence enhancement of arch neuronal optogenetic reporters
Source: Nat Commun. 2022 Oct 28;13:6432. doi: 10.1038/s41467-022-33993-4 (PMC9616920; doi:10.1038/s41467-022-33993-4)
Supplement: Supplementary file 1 — Supplementary Information [file 41467_2022_33993_MOESM1_ESM.pdf]

Supplementary Information

On the Fluorescence Enhancement of Arch Neuronal Optogenetic Reporters

[1]Dipartimento di Biotecnologie, Chimica e Farmacia, Università di Siena; via A. Moro 2, I-53100 Siena, Italy.

[2]Department of Chemistry, Bowling Green State University; Bowling Green, OH 43403, United States.

[3]Institut de Chimie Radicalaire (UMR-7273), Aix-Marseille Université, CNRS, 13397 Marseille, Cedex 20, France.

[4]Department of Chemistry, Kyungpook National University, Daegu 702-701, South Korea.
[5]University of Strasbourg Institute for Advanced Studies; 5, alleé duGeń eŕ al Rouvillois, F-67083 Strasbourg, France.

*Correspondence to: olivucci@unisi.it
† Present address: University of Durham, Department of Chemistry; South Road, Durham DH1 3LE, United Kingdom.

‡ Present address: Dipartimento di Chimica, Università di Pisa; Via Giuseppe Moruzzi, 13, I-56124 Pisa, Italy.

This PDF file includes:

Supplementary Methods 1 - 11
Supplementary References (1-40)
Supplementary Tables 1 - 11
Supplementary Figures 1 - 18

Supplementary Methods 1. Comparative modelling of the Arch3 variants

The crystal structure of the wild type microbial rhodopsins Arch2 and Arch3 are available in the Protein Data Bank[1], with PDB ID 3WQJ (1.8 Å resolution) and 6GUX (1.3 Å resolution), respectively [2,3]. Arch2 and Arch3 share a sequence identity of ca. 86% (**Supplementary Figure 1**). QuasAr1, Archon2, QuasAr2, Arch7 and Arch5 are Arch3-based mutants developed by experiments of site-directed mutagenesis [4–6]. QuasAr1 and QuasAr2 differ from their progenitor for 5 mutations. The P60S, T80S, D106H and F161V mutations are shared by QuasAr1 and QuasAr2, whereas at position 95 the aspartic acid of Arch3 is substituted by a histidine (D95H) in QuasAr1 and by a glutamine in QuasAr2 (D95Q). Arch5 also differs for 5 mutations from Arch3. These are D95E, T99C, V59A, P60L and P196S. Finally, Arch7 has all Arch5 mutations plus D222S and A225C, yielding 7 mutations in total with respect to Arch3. Finally, Archon2 differs from Arch3 for the substitutions T56P, P60S, T80P, D95H, T99S, T116I, F161V, T183I, L197I, A225C. All mutations are shown in **Supplementary Figure 2**.

Since no crystallographic structure has been reported to date, for any of the 5 mutants, comparative modelling was employed for the construction of the corresponding three-dimensional structures and employed for subsequent *in silico* QM/MM model building. The strategy employed exploits the high sequence identity (> 97% with no gaps in the alignment, see also **Supplementary Figure 1**) and better (with respect to Arch2) resolution of the crystal structure of Arch3 as the template. Comparative modeling was performed with the software MODELLER[7]. The structures of the targets were generated by simply copying the coordinates of all the conserved amino acids from the template to the new structures and relaxing the atom coordinates of the mutated side chains. The crystallographic water molecules were also transferred from the template. The relaxed cartesian coordinates of the mutated side-chains are

randomized and then refined by MODELLER via cycles of molecular dynamics (MD) and conjugate gradient (CG) optimizations to minimize an objective function derived from spatial restraints [7]. These operations are repeated 200 times using the different seeds produced by the randomization step to yield 200 potential target structures differing in mutated side-chain conformation. The structures are then scored with the discrete optimized protein energy (DOPE) statistical potential[8]. The final representative variant model is selected as the one featuring the lowest DOPE score.

Supplementary Methods 2. QM/MM model construction and application

2.1 The *a*-ARM protocol

The QM/MM models of the Arch3, Arch2 and of the five Arch3 variants QuasAr1, Archon2, QuasAr2, Arch7 and Arch5 (from now on called the Arch set) were automatically generated using the *a*-ARM protocol developed in the author lab. [9,10]**.** This protocol allows for a relatively fast and standardized construction of a monomeric "gas phase" hybrid quantum mechanical/molecular mechanical (QM/MM) models of rhodopsins and its mutants. The *a*-ARM target is to deliver a basic *in silico* representation of complex protein systems which reproduce trends in spectral and photochemical properties. The resulting *a*-ARM model can be described as a three subsystem (or layer) model (see **Supplementary Figure 3**):

- **QM subsystem** (QM atoms): includes the retinal chromophore atoms, the linker Lysine (K226) side chain atoms starting from the N-terminal to the C_δ and the hydrogen link atom (HLA).
- **MM cavity subsystem** (MM relaxed atoms): includes the MM atoms which are free to relax during the *a*-ARM molecular dynamics (MD). These are the cavity residues, water

molecules within 4Å of the chromophore and the linker-lysine atoms (from C_γ to C_α) which are not QM atoms.

- **MM frame subsystem** (protein frozen atoms): all the protein, ion and water molecule atoms which are not already included in the first two subsystems.

The *a*-ARM protocol takes as input PDB files corresponding either to crystal structures from the PDB or comparative models. The program then identifies ionizable residues, predicts their protonation states and calculates the global charge of the protein. The system, if necessary, is then neutralized by adding Na^+ and Cl^- ions at the intracellular (IS) or extracellular (OS) side of the protein. The residues surrounding the retinal (also called cavity residues) identify the MM cavity and the three computational subsystems mentioned above are created accordingly. The MM frame allows to retain in the structural information from the crystallography structure (as in the case of Arch2 and Arch3) or from the comparative models (as in the case of QuasAr1, Archon2, QuasAr2, Arch5 and Arch7).

Once the above setup is complete, hydrogen atoms are added to the whole protein and their position is concurrently optimized. At this point, the MM cavity atoms and QM atoms are equilibrated via an MM energy minimization followed by parallel MM molecular dynamics runs (N=10 repetitions starting with 10 different seeds) of 1 ps. The generated 10 models are then prepared for successive QM/MM model building. The HLA is positioned at the QM/MM frontier (**Supplementary Figure 3**). The 10 models are subsequently optimized through sequential steps featuring an increasing level of theory (from HF/3-21G/AMBER to single state CASSCF (12,12)/6-31G*/AMBER), to obtain 10 equilibrated geometries. These geometries are used to calculate 10 vertical excitation energies ($\Delta E^{a,\ a\text{-ARM}}_{S1\text{-}S0}$) at the CASPT2 level over a three-root state average SA3-CASSCF (12,12)/6-31G*/AMBER zeroth-order wavefunction (in short, SA3-CASSCF/AMBER level) which we indicate as CASPT2/SA3-CASSCF (12,12)/6-31G*/AMBER level (in short, CASPT2/AMBER level). The average of the 10 resulting values is compared to

the experimentally observed $\Delta E^{a,Exp}_{S1\text{-}S0}$ (see **Supplementary Table 1** for a summary of the relevant experimental spectroscopical data).

2.2 Absorption and emission maxima

We start by defining the procedure adopted to compute. $\Delta E^{a,\, a\text{-}ARM}_{S1\text{-}S0}$, the corresponding emission energy $\Delta E^{f,\, a\text{-}ARM}_{S1\text{-}S0}$ and the energy profile along the reaction path. After the construction of the 10 a-ARM replicas and calculation of the corresponding $\Delta E^{a,\, a\text{-}ARM}_{S1\text{-}S0}$ values (see above), we select, for each member of the Arch set, the replica with the $\Delta E^{a,\, a\text{-}ARM}_{S1\text{-}S0}$ value closest to the average. For that replica $\Delta E^{a,\, a\text{-}ARM}_{S1\text{-}S0}$ is recomputed at the XMS-CASPT2/SA3-CASSCF (12,12)/ANO-L-vDZP/AMBER level of theory (in short, XMS-CASPT2/AMBER level). The corresponding results are reported in **Supplementary Table 2** together with those computed at the CASPT2/AMBER level, which is the level used to benchmark the *a*-ARM protocol [9,10]. Comparison of the mean absolute error (MAE) and mean absolute deviation (MAD) the trend deviation (|| Trend. Dev ||) obtained at such level (for definition see **Supplementary Table 2**) with those obtained at the mentioned XMS-CASPT2/AMBER level show similar values. Notice that the $\Delta E^{a,\, XMS}_{S1\text{-}S0}$ values obtained at the XMS-CASPT2/AMBER level is also re-computed *after geometrical corrections* as described in**.** *Below and in the main manuscript we refer exclusively to the final excitation energy values obtained after applying the geometrical correction.*

The *a*-ARM model of wild-type Arch3 is a reasonable template as it reproduces the experimental value of maximum absorption wavelength ($\lambda^{a,Exp}_{max}$ = 556 nm; $\Delta E^{a,\, Exp}_{S1\text{-}S0} = hc/\lambda^{a,Exp}_{max}$ = 51.4 kcal mol^{-1}). As shown in **Supplementary Table 2**, the computed $\Delta E^{a,\, XMS}_{S1\text{-}S0}$ displays a deviation from $\Delta E^{a,\, Exp}_{S1\text{-}S0}$ of +2.9 kcal mol^{-1} ($\Delta\Delta E^{a,\, Exp,XMS}_{S1\text{-}S0}$), which is within the 4.0 kcal mol^{-1} (blue shifted) established error-bar of the *a*-ARM protocol. Same reasoning applies to the other wild-type

rhodopsin of the Arch set (Arch2), whose QM/MM model features a $\Delta\Delta E^{a,\ Exp,XMS}_{S1\text{-}S0}$ of +3.0 kcal mol^{-1}. The variant models (QuasAr1, Archon2, QuasAr2, Arch7, Arch5) generated automatically using exactly the same protocol are expected to display a similar quality. In fact, the experimental trend in vertical excitation energies is reproduced with a systematic blue-shift with respect to the experimental values (based on the $\lambda^{a,Exp}_{max}$). In fact, all models feature a $\Delta\Delta E^{a,\ Exp,XMS}_{S1\text{-}S0}$ < 4.0 kcal mol^{-1}, and the $\Delta E^{a,Exp,XMS}_{S1\text{-}S0}$ are found to display the same ordering as the experimental $\Delta E^{a,\ Exp}_{S1\text{-}S0}$. Other indicators of the quality of the *a*-ARM models are the MAE and MAD of || Trend. Dev ||. Here, the performance of the Arch set is of 3.1 kcal mol^{-1} for the MAE and kcal mol^{-1} which is also within error bars of *a*-ARM.

The *a*-ARM models were employed to calculate the vertical excitation energy ($\Delta E^{f,\ XMS}_{S1\text{-}S0}$) corresponding to the wavelength of maximum (fluorescence) emission ($\lambda^{f,\ Exp}_{max}$) to be compared with the available experimental values. $\Delta E^{f,\ XMS}_{S1\text{-}S0}$ was estimated as the energy difference between S_1 and S_0 at the S_1 minimum (FS, see main text) obtained by running an S_1 geometry optimization at the SA2-CASSCF (12,12)/6-31G*/AMBER level (in short SA2-CASSCF/AMBER level) starting from the vertically excited Franck-Condon (FC) point. Notice that, in the present contribution we focus on the fluorescence arising from such point. This is assumed to be the source of one-photon induced fluorescence or, equivalently, the fluorescence of the dark-adapted (DA) state of the protein. The corresponding experimental $\lambda^{f,\ Exp}_{max}$ values are available only for the Arch variants QuasAr1, Archon2, QuasAr2, Arch7, Arch5, but not for the wild-type Arch2 and Arch3 (for Arch3 the observed low FQY) is from a photocycle intermediate different from the DA state and requires the absorption of three-photons to be kindled. As shown in **Figure 3A** and reported in **Supplementary Table 3**, the models reproduce the observed trend in $\lambda^{f,\ Exp}_{max}$. However, the $\Delta\Delta E^{f,\ Exp,XMS}_{S1\text{-}S0}$ difference between computed and observed quantities is larger in

absolute value and opposite in sign with respect to $\Delta\Delta E_{S1\text{-}S0}^{a,\,Exp,XMS}$ leading to a relatively large Stokes shift error. However, it should be noticed that the estimation of $\Delta E_{S1\text{-}S0}^{f,\,XMS}$ from simple energy minimization does not correctly handle a non-equilibrium situation (i.e. it does not account for kinetic energy not yet redistributed). To account for such a discrepancy, we have scaled the computed data using a scaling factor reported in ref. [11] where it was found, based on excited state trajectory calculations on different microbial rhodopsins, that the excess kinetic energy yields blue shifted values of ca. 5 kcal mol^{-1}.

Supplementary Methods 3. Correlation between fluorescence quantum yield and photoisomerization barrier

In the main text we demonstrate a correlation between quantities *computed* using the QM/MM models defined above and the *observed* fluorescence quantum yield (FQY) of the Arch set. The FQY expresses how efficiently a molecule converts an absorbed photon into an emitted photon:

$$\mathrm{FQY} = \frac{\mathrm{No.\,of\,emitted\,photons}}{\mathrm{No.\,of\,absorbed\,photons}} \qquad (1)$$

and is a function of the relative rate of radiative and non-radiative de-activation processes:

$$\mathrm{FQY} = \frac{\mathrm{k_r^S}}{\mathrm{k_r^S} + \mathrm{k_{nr}^S}} \qquad (2)$$

where $\mathrm{k_r^S}$ is the rate constant for radiative deactivation (τ^S=1/$\mathrm{k_r^S}$ is called radiative lifetime) and $\mathrm{k_{nr}^S}$ accounts for all the possible non-radiative decays of excited state molecules from S_1 to S_0. In the present research, we assume that: (i) $\mathrm{k_r^S}$ is similar in all closely related Archaearhodopsin homologues and (ii) that $\mathrm{k_{nr}^S}$ is determined by the S_1 double-bond isomerization barrier (E^f_{S1}) of the chromophore. If i-ii hold, it is then possible to hypothesize the existence of a direct proportionality between E^f_{S1} and FQY. More specifically, FQY is modulated by controlling the access to the S_1 double-bond isomerization path leading to the conical intersection (CoIn) region responsible for S_1 decay. This assumption is supported by the τ^S value of virtually all rhodopsins

being estimated in 1-10 ns range[12] by the Strickler-Berg equation[13]. This is several order of magnitude larger than measured τ^{S} even for highly fluorescent rhodopsins, indicating that k_{r}^{S} << k_{nr}^{S} and, as a working hypothesis, a k_{nr}^{S} corresponding to an ultrafast chemical reaction. Under the assumptions i-ii, an Arrhenius model would predict E^{f}_{S1} and FQY to be linked by a logarithmic relationship. However, an Arrhenius model cannot be applied in a non-equilibrium situation such as that of barrierless (or nearly barrierless) isomerization reactions. Accordingly, we do not attempt to fit the E^{f}_{S1} and FQY proportionality relation based on an Arrhenius model but only demonstrate that the FQY and E^{f}_{S1} are directly proportional.

Supplementary Methods 4. Excited state reaction path calculation

To support the existence of a correlation between E^{f}_{S1} and FQY is necessary to compute, for all members of the Arch set, the position of the energy maximum (assumed to correspond to the transition state TS_{S1}) along the S_1 isomerization coordinate using the QM/MM models defined above. Once the TS_{S1} position is known one can compute the E^{f}_{S1} value corresponding to the TS_{S1} - FS energy difference. In the main text, we show that E^{f}_{S1} is connected to the energy difference between the S_1 twisted diradical (TIDIR) intermediate and FS. From now on, this quantity will be referred to as $\Delta E_{TIDIR\text{-}FS.}$

As mentioned in **Supplementary Methods 2**, the *a*-ARM protocol generated N=10 *a*-ARM ground state (S_0) equilibrated structure for each protein. For each member of the Arch set we select the QM/MM model with the vertical excitation energy closest to the average $\Delta E^{a,\,XMS}_{S1\text{-}S0}$. These selected S_0 energy minima correspond to the FC point and are used as starting point for the calculation of the energy profile along the S_1 torsional coordinate of the isomerizing double bond via a relaxed scan (RS) approximation. Since Arch2 and Arch3 are microbial rhodopsins and the 5 mutants are derived from Arch3, the C12-C13-C14-C15 (α) dihedral was assumed to dominate the S_1 isomerization coordinate.

The FC geometry is first re-optimized at the SA2-CASSCF/AMBER level of theory for consistency with the level of theory of the whole reaction path calculations (a justification of the employed QM method and the implementation of a correction are discussed in Section **Supplementary Methods 5**). The obtained FC structure is then used as a guess for the optimization of the S_1 energy minimum that is assigned to the one-photon fluorescent state (FS), via unconstrained optimization at the same level of theory. If an FS cannot be found, the corresponding Arch set member must feature a barrierless path connecting FC directly to TIDIR and/or CoIn. This is the case of the wild-types Arch2 and Arch3 where no FS was identified (**Supplementary Table 4** and **Supplementary Figure 4**).

After FS optimization, the RS is calculated as a relaxed scan along α in the counterclockwise (CCW) direction and starting at FS. Constraints are imposed on α and the other three dihedral angles associated to the C13=C14 bond. For Arch2 and Arch3 lacking a FS the first scan point (-155°, which is reasonably close to the α value of the other models FS, see **Supplementary Table 4**) was used as the RS starting point. In all cases, the resulting RS connects FS to TIDIR (see main text and **Supplementary Figure 4**) and approximate the S_1 minimum energy path (MEP). Notice that when CoIn is not sloped, CoIn and TIDIR coincides. Technically, the scan is performed by constraining the isomerizing torsional angle α and following the "natural" α counterclockwise (CCW) pre-twisting direction. When α approaches the -90° degrees of torsion, a CoIn optimization is started to find the local intersection space ($IS_{S1/S0}$) minimum. The nearby S_1 TIDIR intermediate is easily computed by an unconstrained optimization started from the optimized CoIn. We found that, at the SA2-CASSCF/AMBER level used in the RS calculation, all Arch3-variants (QuasAr1, Archon2, QuasAr2, Arch5 and Arch7) feature a sloped CoIn and a distinct nearby TIDIR. On the contrary, CoIn has a peaked topography in the wild types (Arch2 and Arch3). Therefore, we concluded that the S_1 product reached by RS is the CoIn in Arch2 and Arch3 and is the TIDIR in all variants. As shown in **Supplementary Figure 5** and

Supplementary Figure 6 (where Arch7 is given as example), after electron correlation correction, the product is TIDIR in all cases as CoIn is always sloped.

Supplementary Methods 5. Perturbative correction of the excited state reaction path

The structural information on the Arch-set is obtained at the SA2-CASSCF/AMBER level. Such level of theory has been extensively benchmarked for rhodopsins and represents a compromise between computational cost and accuracy when both ground and excited states need to be treated in a balanced way [14–17]. Thus, the progression of the geometrical coordinates such as the bond length alternation (BLA) and properties such as Mulliken charges, S_0-S_1 oscillator strength along the RS is calculated at that level of theory (**Supplementary Table 5**). However, both the transition energies and energy profile along the RS needs to be computed at an augmented level of theory as the missing dynamic electron correlation energy must be recovered to get quantities comparable with the experimental data. This is usually carried out via single point computations (i.e. assuming a limited effect of the dynamic electron correlation on geometrical structures) using single-state and/or multi-state multiconfigurational second order Møller-Plesset level of theory. In the present work we employ both the intermediate single-state CASPT2/AMBER and advanced multistate XMS-CASPT2/AMBER levels. It has been shown in literature that such level corrects certain artefacts of the CASPT2/AMBER level and of its multi-state (MS-CASPT2) variants, especially when it comes to the description of the region approaching and surrounding the CoIn[18,19].

In contrast with previous studies where the perturbative was performed at the single point level[11,20], here we implemented a geometrical correction accounting for the effects of the dynamic electron correlation energy. Since previous studies on retinal chromophore models, show that BLA is the geometrical coordinate maximally sensitive to such effects, we correct such a

coordinate[16,21–23]. To do so we re-optimize the FC structure at the MP2/6-31G*/AMBER level that includes the electron correlation effects in a situation where the missing non-dynamical electron correlation effect is minimal (i.e., in a closed-shell system). This geometry is then used to identify a BLA "correction vector" as the difference between FC structures computed at the SA2-CASSCF/AMBER and MP2/6-31G* levels respectively. The correction vector spans, almost exclusively, the BLA mode and it is then applied to all points of the RS from FC to TIDIR to get a geometrically and energetically corrected MEP. Such a MEP is constructed by locating S_1 energy minima (on S_0 for the FC point and on S_1 for all remaining points) along the correction vector at the XMS-CASPT2/AMBER level (see **Supplementary Figure 5**). We believe that the described protocol provides the best possible alternative to XMS-CASPT2/AMBER geometry optimizations, which is presently not systematically applicable to QM/MM models due to lack of the XMS-CASPT2/AMBER gradients. The applied geometrical correction turned out to be approximately constant along the set.

Notice that, Marin et al. found in a blue-shifted ASR mutant that a near-degeneracy situation of the S_1 and S_2 states was likely to be the origin of a barrier along the S_1 isomerization path [11]. In contrast, as shown in the perturbatively corrected paths of **Supplementary Figure 7,** in the investigated Arch-set, S_1 and S_2 are far from degenerate (i.e., more than 15 kcal mol^{-1} apart along the entire reaction path), and S_2 appears not to be involved in the fluorescent mechanism of our Arch-set. For these reasons, in the main text, we discuss the fluorescence mechanism and the dynamic of the excited state by referring exclusively to the S_1 and S_0 states.

To corroborate the validity of our BLA "correction vector", we re-optimized the geometries of the SA2-CASSCF/AMBER S_1 stationary points (FS and TIDIR) using the state-interaction state-averaged spin-restricted ensemble-referenced Kohn–Sham method (SI-SA-REKS)[24] to treat the QM moiety. The method has been benchmarked on a retinal chromophore model ground and

excited reaction paths documenting an accuracy similar to wavefunction-based multi-state multiconfigurational methods[25]. Notice that, XMS-CASPT2 geometry optimizations at the QM/MM level are still unpractical and therefore we employ REKS for geometry optimization. In fact, the SI-SA2-REKS(2,2) approach that we employed (REKS/AMBER in short), two active electrons in two orbitals are used to describe the π-π* excitation which is main determinant of the RPSB chromophore photochemistry[26]. Such active space also accounts for the static electron correlation while the dynamic electron correlation is included by using an exchange-correlation functional. As shown by Martinez et al. in two studies of Channelrhodopsin-2[27] and Bacteriorhodopsin[28], these features make SI-SA-REKS a valuable tool able to describe conical intersections and excited state reaction paths of the RPSB chromophore with performances comparable to other wavefunction-based multi-state multireference methods[29]. In **Supplementary Figure 8** panel A, we show that although the REKS/AMBER calculated $\Delta E_{TIDIR-FS}$ on the Arch-set is overestimated of few kcal mol^{-1} with respect to the XMS-CASPT2/AMBER method, the error is systematic, resulting in a parallel trend across the Arch-set. Similar conclusions hold when the energies of the REKS/AMBER optimized geometry are corrected at the XMS-CASPT2//SA2-CASSCF(12,12)/ 6-31G*/AMBER level of theory. In **Supplementary Figure 8** panel B it is demonstrated that in our set of QM/MM models the trend in $\Delta E_{TIDIR-FS,}$ is invariant with respect to the methodology employed to optimize the FS and TIDIR geometries, as shown by the equally accurate linear relationship between reaction energy and FQY.

Supplementary Methods 6. Electronic character of the S_1 PES

To describe how the character of the S_1 state changes along the original (SA2-CASSCF/AMBER) α-driven MEP we followed both the variations in number of unpaired electrons (NUE) on the atoms of α (i.e., C12-C13-C14-C15) and fraction of positive charge on

the C14-C15-N moiety. In Figure 2A and 2B (main text), we report the evolution of the NUE for the zeroth order wavefunction associated to the S_1 relaxed scans for Arch3 (left) and Arch7 (right) using the analysis toolkit Multiwfn [30]. We also reported the sum of the charges on the -C14H-C15H-NH-CH3 (for short C14-C15-N) moiety from the Mulliken Population Analysis. The results are shown in **Supplementary Figure 4** (charge) and **Supplementary Figure 9** (NUE). We evaluated the same properties after applying the correction vector (see above) to the MEP (see **Supplementary Methods 5** for details), to check whether they qualitatively agreed with the SA2-CASSCF/AMBER results. To verify the quality of the recently implemented XMS-CASPT2 method in [Open]Molcas v19.11 [31], we re-performed the analysis with the equivalent but robust XMCQDPT2 method implemented in Firefly v8.2 [32]. Notice that, in the last case the analysis employs the first order QDPT densities calculated at the XMCQDPT2/SA3-CASSCF/cc-pVTZ level of theory (in short XMCQDPT2/AMBER) and including the AMBER charges (in short XMCQDPT2/$AMBER_{charges}$) of the corresponding MEP structures. As discussed in the main text, the computed variations of the S_1 electronic character appears to be relatively insensitive to the QM level of theory employed. The corresponding data are reported in **Supplementary Table 6** (free valences at the FC, FS and TIDIR geometries), **Supplementary Table 7** (bond order analysis at the same geometries). Finally, **Supplementary Figure 7** shows the Mulliken charge along the geometrically corrected reaction path, while in **Supplementary Figure 10** we report the evolution of the NUE.

Supplementary Methods 7. Conical intersection topography.

To complement the computed MEPs, we plot the S_0 and S_1 energies along the branching plane (BP) of the optimized CoIn for the SA2-CASSCF/AMBER models of Arch3, QuasAr1 and Arch7. Notice that we did not perform such calculations at the XMS-CASPT2/AMBER level since the

QM/MM XMS-CASPT2 gradient needed to optimize CoIn structures are not currently available. The plot is generated using the orthogonalized branching plane (BP) vectors (i.e. the orthogonalized gradient difference, $\boldsymbol{X_1} = \frac{\partial(V_{S1}-V_{S0})}{\partial R}$, and derivative coupling, $\boldsymbol{X_2} = \langle\psi_{S0}|\frac{\partial}{\partial R}|\psi_{S1}\rangle$ where V_{S1} and V_{S0} are the Born-Oppenheimer (adiabatic) potential energy surfaces ψ_{S0} and ψ_{S1} are the corresponding wavefunctions. In **Supplementary Figure 11** we display the components of these BP vectors on the retinal atoms (the most significant contribution to $\boldsymbol{X_1}$ and $\boldsymbol{X_2}$ were scaled up by a factor 2). It is apparent that the dominant components are the C14-C15 and C15=N bond stretches ($\boldsymbol{X_1}$) and the twisting of the reactive C12-C13=C14-C15 double bond ($\boldsymbol{X_2}$) and correlate with the MEP driving torsional coordinate α.

Apart from providing insights into the topographical properties, the BP analysis allows to visualize the charge distribution and, therefore, the electronic character of S_1 and S_0 in the CoIn region. Panels A, C, E of **Supplementary Figure 12** shows the fraction of positive charge located on the C14-C15-N moiety that is proportional to the $1A_g$ covalent/diradical (COV/DIR) character and inversely proportional to the $1B_u$ charge-transfer (CT) character. Loosely, the electronic character is conveniently assigned based on the total charge residing on the -C14H-C15H-NH-CH3 (for short C14-C15-N) moiety of the chromophore (i.e. QM subsystem). A full COV/DIR character would feature a moiety hosting a >80% positive charge. In contrast, a full CT character would correspond to a moiety with a <20% positive charge. The analysis of the results allows to enforce the conclusion (already suggested by the unconstrained optimization starting from the CoIn geometry discussed in **Supplementary Methods 4**) that the sloped CoIn structures of the fluorescent Arch3 mutants are connected to an exotic intermediate (TIDIR) by the BP vector describing the BLA mode.

Supplementary Methods 8. Two-state two-mode model of the Arch PESs

In this section we present an analytical two-state (S_0 and S_1) and two-modes (α and BLA) diabatic model reproducing the S_1 and S_0 PES topographical modifications showed by the Arch proteins. Most relevantly, the model reproduces the Arch2 to Arch7 increasing energy difference between TIDIR and FS minima as well as the changes in the corresponding E^f_{S1} barrier and TS_{S1} position (x^f_{S1}). The model is a modified and refitted version of a previously reported Hamiltonian for the minimal retinal chromophore (molecular) model PSB3 [33]. In the diabatic representation the two-mode PESs assume the form:

$$\widehat{H}_{el}(\alpha,r) = \begin{pmatrix} Hcov2D(\alpha,r) & Hcp(\alpha,r) \\ Hcp(\alpha,r) & Hct2D(\alpha,r) \end{pmatrix} \quad (3)$$

Where *Hcov2D* is the potential energy of a diabatic state describing the COV/DIR character (also called $1A_g$) while *Hct2D* is the potential energy of a diabatic state describing the CT character (also called $1B_u$). *Hct2D* represents the electronic coupling between the two diabatic states. These matrix elements are function of the geometrical parameters α and r correspond to the α and BLA coordinates of the Arch models. The parametrization of *Hcov2D, Hct2D* and *Hct2D* (see the parametrization coefficients to be determined below) is based on the computational results carried out at the XMS-CASPT2/AMBER level. The diabatic states corresponding to *Hcov2D* and *Hct2D* have been defined in such a way to (qualitatively) reflect the computed changes in total positive charge residing on the C14-C15-N moiety and, therefore, follow the changes in the chromophore electronic character (see above). Their expressions are:

$$Hct2D(\alpha,r) = \left(1 + c_5 \sin\left[\frac{\pi\alpha}{180}\right]\right)^2 (c_1 r^2 + c_2 r + c_3) + c_4 \cos\left[\frac{\pi\alpha}{180}\right]^2 \quad (4)$$

$$Hcov2D(\alpha,r) = \sin\left[\frac{\pi\alpha}{180}\right]^2 (Morse_1(r) + d_2) + d_3 \cos\left[\frac{\pi\alpha}{360}\right]^2 + Morse_2(r) \cos\left[\frac{\pi\alpha}{360}\right]^2 \quad (5)$$

$$Hcp(\alpha) = k_1 \, sin\left[\frac{\pi\alpha}{90}\right] \quad (6)$$

In Eq. (5) the Morse potentials are:

$$Morse_1(r) = 2000 \times (-1 + e^{-d_1(r-0.02508)})^2 \quad (7)$$

$$Morse_2(r) = 2000 \times (-1 + e^{-d_4(r-0.09126)})^2 \quad (8)$$

while $d_1 = \sqrt{kf_1/4000}$ and $d_4 = \sqrt{kf_1/4000}$ (see the meaning of these parameters in ref. [33]). To reproduce the XMS-CASPT2/AMBER topographical changes along the Arch set, $\widehat{H}_{el}(\alpha, r)$ has been adjusted in the following way:

(i) $Hcov2D(\alpha, r)$, $Hcp(\alpha)$, $Hct2D(\alpha, r)$ have been scaled by a factor $f = 1.87$. f is calculated as $f = \frac{95.7 \, kcal \, mol^{-1}}{51.2 \, kcal \, mol^{-1}}$; where 95.7 kcal mol^{-1} and 51.2 kcal mol^{-1} are the excitation energy at the FS geometry for PSB3 [33] and Arch2 respectively. Therefore, Equation (3) becomes:

$$\widehat{H}_{el}(\alpha, r) = \begin{pmatrix} Hcov2D(\alpha, r)/f & Hcp(\alpha, r)/f \\ Hcp(\alpha, r)/f & Hct2D(\alpha, r)/f \end{pmatrix} \quad (9)$$

(ii) $Hcp(\alpha, r)$ is multiplied by the factor u = 1.45.

$$Hcp(\alpha, r) = Hcp(\alpha, r) * u \quad (10)$$

The parameter u has been chosen to simulate the computed E^f_{S1} barrier changes along the Arch series. Since the diabatic energies $Hct2D$ and $Hcov2D$ are closer than PSB3, it is reasonable to increment the electronic coupling between them.

(iii) To reproduce the stabilization of FS along the Arch set, $Hct2D$ has been reformulated as:

$$Hct2D(\alpha, r) = \left(1 + c_5 sin\left[\frac{\pi\alpha}{180}\right]\right)^2 (c_1 r^2 + c_2 r + c_3) + (c_4 + f_{CT} \cdot n) \, cos\left[\frac{\pi\alpha}{180}\right]^2 \quad (11)$$

f_{CT} accounts for the stabilization of the CT diabatic state and consequently the lowering of the S_1 - S_0 energy gap at FC and FS. It is worth noticing that $cos\left[\frac{\pi\alpha}{180}\right]^2$ make the f_{CT} term vanish along $\alpha = k\,\pi/2$ (see **Figure 3D** of the main text).

(iv) To reproduce the destabilization of TIDIR, the $Hcov2D$ expression was changed to:

$$Hcov2D(\theta,r) = sin\left[\frac{\pi\alpha}{180}\right]^2 (Morse_1(r) + d_2 + f_{COV} \cdot n) + d_3 cos\left[\frac{\pi\alpha}{360}\right]^2 + Morse_2(r) cos\left[\frac{\pi\alpha}{360}\right]^2 \quad (12)$$

f_{COV} accounts for the de-stabilization of the COV/DIR diabatic state and consequently the increasing of S_1 - S_0 energy gap at TIDIR. It is worth noticing that $sin\left[\frac{\pi\alpha}{180}\right]^2$ makes the f_{COV} term vanish along $\alpha = k\,\pi$. In addition, the parameter d_2, tuning the TIDIR energy, has been set to be:

$$d_2 = 48.0 * f - d_3 cos\left[\frac{\pi\alpha}{360}\right]^2 \quad (13)$$

Where f has been defined in (i) and 48.0 kcal mol^{-1} is the S_1 energy with respect to FC at the TIDIR geometry for Arch2 (see **Supplementary Table 5**), f_{CT} and f_{COV} are computed as:

$$f_{CT} = (51.2 - 48.6)\ /\ \mathrm{m} \quad (14)$$

$$f_{COV} = (54.8 - 48.0)\ /\ \mathrm{m} \quad (15)$$

Where 51.2 and 48.6 kcal mol^{-1}are the vertical excitation energy at FS of Arch2 and Arch7, respectively while 54.8 and 48.0 kcal mol^{-1} are the excitation energies at TIDIR or Arch2 and Arch7, respectively.

In **Supplementary Figure 13** we show the PESs computed assuming m = 3 and n = - 1, …, 6. These two parameters can be chosen in such a way to reproduce the properties of the Arch set. m sets the "spacing" between two contiguous points in the series; the larger is m, the smaller is the variation of $Hct2D(\alpha,r)$ and $Hcov2D(\alpha,r)$. n represents the index along the Arch series. With n = 0, f_{CT} and f_{COV} produce no modification of the diagonal element of $\widehat{H}_{el}(\alpha,r)$; This setup

provides the best possible fit of Arch2's PES. Incrementing n, the modifications become larger, consistently with points (iii) and (iv). More specifically, n = 6 is associated with the maximum modification of the two diabatic surfaces resulting in a $\Delta E_{TIDIR-FS}$ value comparable with the computed value of Arch7. For the sake of completeness, we report the result also with n = -1 corresponding to a hypothetical protein in which the $\Delta E_{TIDIR-FS}$ value is lower than the value computed for Arch2.

Supplementary Methods 9. Excited state trajectory calculations

Excited state trajectories are used to complement the "static" MEP information and introduce possible kinetic energy effects in the system. To assess whether the reaction paths (see Section **Supplementary Methods 4** and **Supplementary Methods 5**) provide meaningful information (e.g., on the S_1 isomerization barriers), we have probed the S_1 dynamics of Arch3, QuasAr1, Arch7 as representative models of the Arch set. This has been done by propagating FC trajectories at the SA2-CASSCF/AMBER level on S_1 and staring at the consistently optimized FC structure. These are deterministic trajectories propagated from the FC point on S_1 with zero initial velocities, which approximate the average evolution of multiple trajectories with a Boltzmann-like distribution of initial velocities (i.e. the center of the vibrational wavepacket assumed to remain compact) [34]. All calculations were carried out using the MOLCAS v8.1/TINKER package [35] at the SA2-CASSCF/AMBER level (XMS-CASPT2/AMBER gradients not being available) .

Supplementary Figure 14 and **Supplementary Figure 15** show that Arch3 decays to S_0 in the CoIn region on a sub-500 fs timescale after propagating mainly along α. This is consistent with a negligible E^f_{S1} value consistently with the corresponding approximated MEP. In contrast, the S_1 time evolution of QuasAr1 and Arch7 (center and right column in **Supplementary Figure 14**) show that these variants orbit in the FS region for the entire simulation with only limited α

progression. In these cases, the larger E^f_{S1} (**Supplementary Figure 4**) must be capable to slow down the isomerization.

The FC trajectories also provide information on the time evolution of the electronic character. In all representative cases a substantial change occurs within 20 fs, where the dominating character inverts from $1B_u$ to $1A_g$ (**Supplementary Figure 14**, second row) and then display oscillations of the S_1 charge perfectly coupled with those of S_0 charge. Furthermore, the bottom row of **Supplementary Figure 14** shows how these oscillations couple with BLA coordinate changes.

Supplementary Methods 10. Analysis of the E^f_{S1} and reaction energy in terms of electrostatic and steric effects

10.1 *In vacuum* analysis

As shown in Figure 4A of the main text, it is evident that the opsin environment has a remarkable impact on the E^f_{S1} magnitude across the Arch-set. In particular, the comparison of the S_1 energy profiles of Arch3 and Arch7 computed in absence (*in vacuum)* and presence of the protein environment, indicates that the environment is responsible for the Arch3 barrierless MEP. In the profiles calculated *in vacuum*, the chromophore geometry was kept frozen at the position found in the corresponding geometrically corrected XMS-CASPT2/AMBER approximated MEP (Section **Supplementary Methods 5**). While in the main text only the comparison between Arch3 and Arch7 is reported, in **Supplementary Figure 16** we also show the *in vacuum* profile of QuasAr1. It is evident that removing the opsin interactions produces similar S_1 energy profiles for all the three models. More specifically, in absence of the protein Arch3, QuasAr1 and Arch7 have a 4.6, 3.2 and 3.1 kcal mol^{-1} isomerization barriers (E^f_{S1}), respectively (**Supplementary**

Table 9). This suggests that the opsins impose geometrically similar chromophore isomerization coordinates. Therefore, we suggest that it must be the protein environment, mainly via the cavity amino acids, to determine a strong stabilization of TIDIR with respect to the FS in Arch3 (with a consequent decrease in E^{f}_{S1}). It needs to be established if such stabilization has a steric or electrostatic origin.

10.2 Static van der Waals analysis

For this reason, we re-calculated the XMS-CASPT2/AMBER profiles in the absence of electrostatic effects (i.e. by setting the MM point charges of the protein to zero) but leaving the van der Waals interactions unaltered, keeping the chromophore geometry frozen. **Supplementary Figure 17** shows the result of such analysis (extended data at **Supplementary Table 10**). The resulting S_1 energy profiles are almost perfectly overlapping with those calculated *in vacuum* and all of them display a significant barrier with E^{f}_{S1} values of 4.4, 5.2 and 6.2 kcal mol^{-1} for Arch3, QuasAr1 and Arch7, respectively. This supports the conclusion that while the barrier originates from opsin-imposed geometrical constraints, the electrostatic effects are responsible for the energy barrier modulation across the Arch-set.

10.3 Geometrical effects of the van der Waals interactions

As a final test to support the conclusions already discussed in **Supplementary Methods 10.2** and in the main text**,** we re-calculated an approximated MEP in presence of van der Waals interactions only (MEP_{VdW}), this time allowing the geometrical relaxation of the cavity sidechains and of the retinal chromophore. Since XMS-CASPT2/AMBER geometry optimizations could not be performed, MEP_{VdW} was computed at the SA2-CASSCF/AMBER level, by setting to zero the MM point charges. Similarly to what has been documented in **Supplementary Methods 10.2**,

the computed MEP_{VdW} is, again, similar for Arch3, QuasAr1 and Arch7 (**Supplementary Figure 18** and **Supplementary Table 11**). Indeed, all the three models feature a barrier along the S_1 isomerization path (4.2 kcal mol^{-1} for Arch3, 3.1 kcal mol^{-1} for QuasAr1 and 3.2 kcal mol^{-1} for Arch7). This final result enforce the conclusion that steric (van der Waals) interactions are “shaping" the computed reaction coordinates similarly across the Arch-set, but E^f_{S1} must be regulated by the differential electrostatics observed in the different QM/MM models.

10.4 ESP cross-section

To assess the effect of the opsin electrostatic potential (ESP_{opsin}) on the MEP energy profile, we calculated the Coulombic component of the electrostatic potential generated by the MM point charges of the protein at FS and TIDIR for all members of the Arch set. Since we are particularly interested in evaluating the differences in ESP_{opsin} projected by the opsin onto the retinal, we evaluated such differences on a bi-dimensional cross-section cutting through the π-system (C5 to N) of the chromophore. To do so, we defined least squares fit plane (ordinary least squares fitting procedure) using the heavy atoms coordinates of the retinal chromophore backbone as the set of 3D points to be fitted. We then defined a grid of points of dimensions 16Å x 6Å (containing the retinal chromophore) on such plane at which we evaluated the ESP generated by the opsin point charges. The potential at the j-th grid point is defined as:

$$\boldsymbol{V_j} = \sum_{i}^{N} \frac{q_i}{r_{ij}} \qquad j = 1,2,\ldots,M; \tag{16}$$

where j runs over the M grid points and i runs over the N point charges of the opsin. The point charges were taken from the AMBER94 Force Field consistently with the MM point charges used in the *a*-ARM model. For the sake of comparison between different proteins (see main text), to define the grid of points for the ESP_{opsin} evaluation we used the coordinates of atoms belonging

to the π-systems of the geometries we wished to compare as target for the least squares fitting procedure. These are the same atoms for all the Arch models.

Supplementary Methods 11. Optimization of the cavity electrostatics

Starting from the QM/MM model of Arch3, we designed an optimization allowing the negative charge hosted by the main counterion (in this case the residue D222) to relocate and distribute on the other cavity residues to produce a specific value of $\Delta E_{TIDIR\text{-}FS}$. Briefly, we start from the QM/MM structures of the FS and TIDIR of Arch3, select a target $\Delta E^*_{TIDIR\text{-}FS}$ value for the optimization and optimize the electrostatics of the cavity such that the difference between the absolute values of $\Delta E_{TIDIR\text{-}FS}(\mathbf{q})$ and $\Delta E^*_{TIDIR\text{-}FS}$ is minimized. To achieve this goal, the total charges of the cavity residue of the Arch3 QM/MM model are represented by their MM force field charges per residue (q_i), which is 0 for neutral residues and +1 or -1 for charged residues. Conveniently, in Arch3 QM/MM model, the determined cavity (see **Supplementary Methods 2**), does not include positively charge residues, such that we can represent the model (virtual) counterion charge distribution by a vector ($\mathbf{q}$ = q_0, q_1, …, q_N, where N is the number of residues), whose elements can host a negative charge comprised between 0 and -1. Furthermore, we impose that the global charge of the cavity ($\mathbf{q}$) must always equal -1 (as in the starting QM/MM model), to make sure that the final optimized model is as realistic as possible. Given this set of rules, the problem of finding a $\Delta E^*_{TIDIR\text{-}FS}$ value, can be formulated as a constrained optimization problem as follows:

$$\min\left(\left(\Delta E_{TIDIR-FS}(\mathbf{q}) - \Delta {E^*}_{TIDIR-FS}\right)^2\right) \quad (17)$$

$$\text{subject to} \sum_{i}^{N} q_i = -1\,;\; q_1, q_2, \ldots, q_N \le 0$$

When a residue hosts a fraction of negative charge different from 0 such fraction is equally distributed amongst the atom of the residues. Since $\Delta E_{TIDIR\text{-}FS}$ is calculated at the SA2-CASSCF/AMBER level and no gradient of the CASSCF wavefunction is available with respect to the charges, the minimization is performed by computing the gradient numerically, with the following two-point formula:

$$\frac{(\Delta E_{\mathrm{TIDIR-FS}}(\mathbf{q}) - \Delta E^{*}{}_{\mathrm{TIDIR-FS}})^2 - (\Delta E_{\mathrm{TIDIR-FS}}(\mathbf{q}+\Delta\mathbf{q}) - \Delta E^{*}{}_{\mathrm{TIDIR-FS}})^2}{\Delta\mathbf{q}} \qquad (18)$$

where $\Delta\mathbf{q}$ is set to 0.001. The optimization is carried out at fixed geometry and all the $\Delta E_{TIDIR\text{-}FS}(\mathbf{q})$ are therefore evaluated via single point calculations. Since $\Delta E_{TIDIR\text{-}FS}$ is a reaction energy, for all tested (**q**) two single point calculations needs to be performed, at FS and TIDIR geometries. The optimization of the charge distribution is performed using python Scipy[36] code interfaced with MOLCAS, which performs QM/MM calculation and computes the gradient which is then used by the trust region algorithm[37] implemented in Scipy to perform the minimization and find optimal (**q**) according to Eq. 17.

In the main text we discuss the application of this procedure to target the $\Delta E_{TIDIR\text{-}FS}$ of the brightest mutant Arch7. The algorithm presented is composed of two parts. Part i consists of the (**q**) optimization procedure discussed above. Along the optimization, only (**q**) is relaxed and the geometries are kept fixed. In part ii, once a solution (**q**) has been found, the QM/MM geometries of FS and TIDIR in the new electric field of the charges (**q**) are relaxed on the S_1 PES. At this points, $\Delta E_{TIDIR\text{-}FS}$ is recomputed. At the end of parts i and ii, if $\Delta E_{TIDIR\text{-}FS}$ is

significantly different from $\Delta E^*_{TIDIR\text{-}FS}$, (we consider a tight energy difference threshold of 0.1 kcal mol^{-1}), i-ii are repeated until $\Delta E_{TIDIR\text{-}FS} = \Delta E^*_{TIDIR\text{-}FS}$ according to the selected threshold.

Supplementary Tables

Supplementary Table 1. Summary of relevant available spectral data for Arch2, Arch3 and the engineered Arch3 variants studied in this work.

Protein	$\Delta E^{a,Exp}_{S1\text{-}S0}$ (kcal mol^{-1})	$\lambda^{a,Exp}_{max}$ (nm)	$\Delta E^{f,Exp}_{S1\text{-}S0}$ (kcal mol^{-1})	$\lambda^{f,Exp}_{max}$ (nm)	FQY	Ref.
Arch2	51.5	555	NR[a]	NR[a]	NR	[38]
Arch3	51.4	556	41.6 [b]	687[b]	$1–9 \cdot 10^{-4}$	[4]
QuasAr1	49.3	580	40.0, 38.6	715, 740	$6.5 \cdot 10^{-3}$, $8.0 \cdot 10^{-3}$	[6,39]
Archon2	48.8	586	38.9	735	$1.1 \cdot 10^{-2}$	[5]
QuasAr2	48.5	590	40.0	715	$4.0 \cdot 10^{-3}$	[6]
Arch7	46.4	616	39.3	727	$1.2 \cdot 10^{-2}$	[4]
Arch5	46.0	622	39.1	731	$8.7 \cdot 10^{-3}$	[4]

[a] Not Reported

[b] The λ^{f}_{max} reported is likely coming from the Q-intermediate rather than from the dark-adapted state (see ref. [40])

Supplementary Table 2. Comparison between computed and observed vertical excitation energies for the S_0 equilibrium geometries of the Arch set. The excitation energies reported as $\Delta E^{a,\ a\text{-ARM}}_{S1\text{-}S0}$ were assessed with *a*-ARM CASPT2/AMBER level of theory, while those reported as $\Delta E^{a,\ XMS}_{S1\text{-}S0}$ were calculated at the XMS-CASPT2/AMBER level of theory. All data are in kcal mol^{-1} .

Protein	Experimental		CASPT2/AMBER			XMS-CASPT2/AMBER		
	$\Delta E^{a,Exp}_{S1\text{-}S0}$	$\Delta^{WT,\ Exp(a)}_{max}$	$\Delta E^{a,a\text{-ARM}}_{S1\text{-}S0}$	$\Delta\Delta E^{a,\ Exp,a\text{-ARM}}_{S1\text{-}S0}$	$\Delta^{WT,\ Calc(b)}_{max}$	$\Delta E^{a,\ XMS}_{S1\text{-}S0}$	$\Delta\Delta E^{a,\ Exp,\ XMS}_{S1\text{-}S0}$	$\Delta^{WT,\ Calc(b)}_{max}$
Arch2	51.5	0.0	54.5	3.0	0.0	52.3	0.8	0.0
Arch3	51.4	-0.1	54.3	2.9	-0.2	52.7	1.3	0.4
QuasAr1	49.3	-2.2	53.0	3.7	-1.5	51.3	2.0	-1.0
Archon2	48.8	-2.7	53.0	4.2	-1.5	51.1	2.3	-1.2
QuasAr2	48.5	-3.0	51.8	3.3	-2.7	50.6	2.1	-1.7
Arch7	46.4	-5.1	48.8	2.4	-5.7	48.9	2.5	-3.4
Arch5	46.0	-5.5	48.0	2.0	-6.5	48.2	2.2	-4.1
MAE(c) ± MAD(d) of $\Delta\Delta E^{a,\ Exp,\ a\text{-ARM}}_{S1\text{-}S0}$				3.1 ± 0.6				
MAE(c) ± MAD(d) of $\Delta\Delta E^{a,\ Exp,\ XMS}_{S1\text{-}S0}$							1.9 ± 0.5	
MAE(c) ± MAD(d) of ǁ Trend Dev.ǁ(e)					3.1 ± 1.2			1.9 ± 1.7

[a] Difference between experimental $\Delta E^{a,\ Exp,\ a\text{-ARM}}_{S1\text{-}S0}$ of each model with respect to the experimental value of Arch2 wild-type, taken as reference ($\Delta^{WT,\ Exp}_{max}$).

[b] Difference between the calculated $\Delta E^{a,\ Exp,\ a\text{-ARM}}_{S1\text{-}S0}$ of each model with respect to the calculated value of Arch2 wild-type, taken as reference ($\Delta^{WT,\ Calc}_{max}$).

[c] $MAE = \frac{1}{n}\sum_{i=1}^{n}|y_i - x_i|$, where y is the computational value ($\Delta E^{a,\ a\text{-ARM}}_{S1\text{-}S0}$ or $\Delta E^{a,\ XMS}_{S1\text{-}S0}$) and x is the experimental value ($\Delta E^{a,Exp}_{S1\text{-}S0}$).

[d] $MAD = \frac{1}{n}\sum_{i=1}^{n}|x_i - MAE|$

[e] ǁ Trend Dev. ǁ = | $\Delta^{Wt,\ Exp(a)}_{max}$ - $\Delta^{Wt,\ Calc(b)}_{max}$ |

Supplementary Table 3. Comparison between observed and computed (XMS-CASPT2/AMBER level) fluorescence emission wavelength ($\lambda_{max}^{f, Exp}$) in nanometers for the members of the Arch set displaying fluorescence. The corresponding experimental ($\Delta E_{S1\text{-}S0}^{f, Exp}$) and computed ($\Delta E_{S1\text{-}S0}^{f, XMS}$) energy gap in kcal mol^{-1} are also given. The difference between observed and computed values are given as $\Delta\lambda_{max}^{f}$ and $\Delta\Delta E_{S1\text{-}S0}^{f, Exp, XMS}$.

Model	Experimental		Computational		Error	
	$\Delta E_{S1\text{-}S0}^{f, Exp}$	$\lambda_{max}^{f, Exp}$	$\Delta E_{S1\text{-}S0}^{f, XMS}$	$\lambda_{max}^{f, XMS}$	$\Delta\Delta E_{S1\text{-}S0}^{f, Exp,XMS}$	$\Delta\lambda_{max}^{f, Exp, XMS}$
QuasAr1	39.3	728	35.03	816	-4.3	88
Archon2	38.9	735	32.38	882	-6.5	147
QuasAr2	40.0	715	36.33	787	-3.7	72
Arch7	39.3	727	36.31	787	-3.0	60
Arch5	39.1	731	33.92	843	-5.1	102

Supplementary Table 4. Summary of the most relevant energy data from the MEP computed at the SA2-CASSCF/AMBER level of theory (above) and XMS-CASPT2/AMBER level of theory (below). The FC, FS and TIDIR energies are S_1 energies relative to the corresponding S_0 equilibrium structure (FC).

SA2-CASSCF/AMBER							
**Model**	**FC** (kcal mol^{-1})	**FS** (kcal mol^{-1})	**TIDIR** (kcal mol^{-1})	**$\Delta E_{TIDIR-FS}$** (kcal mol^{-1})	**E^f_{S1}** (kcal mol^{-1})	**x^f_{S1}** (degrees)	(rad)
Arch2	78.05	61.48[a]	48.71[b]	-12.76	0.00	0.0	0.00
Arch3	76.40	59.63[a]	47.53[b]	-12.10	0.00	0.0	0.00
QuasAr1	73.89	59.05	50.18	-8.87	0.64	-135.0	1.37
Archon2	75.18	59.26	55.89	-3.37	2.62	-120.0	2.13
QuasAr2	72.07	59.01	47.00	-12.01	0.33	-135.0	1.40
Arch7	70.79	55.22	52.82	-2.41	1.48	-125.0	1.98
Arch5	66.38	53.37	49.28	-4.09	0.86	-130.0	1.51
**XMS-CASPT2/AMBER**							
**Model**	**FC** (kcal mol^{-1})	**FS** (kcal mol^{-1})	**TIDIR** (kcal mol^{-1})	**$\Delta E_{TIDIR-FS}$** (kcal mol^{-1})	**E^f_{S1}** (kcal mol^{-1})	**x^f_{S1}** (degrees)	(rad)
Arch2	52.29	51.23	48.01	-3.22	0.21	-138.6	0.41
Arch3	52.71	50.51	47.13	-3.38	0.48	-128.9	0.72
QuasAr1	51.32	50.00	49.57	-0.43	2.38	-118.3	0.96
Archon2	51.08	50.29	56.71	6.42	6.42	-98.2	1.57
QuasAr2	50.61	50.32	47.61	-2.71	NA[c]	NA[c]	NA[c]
Arch7	48.92	48.56	54.83	6.27	6.32	-109.2	1.37
Arch5	48.22	47.16	49.93	2.78	3.74	-109.0	1.17

[a]A planar minimum (FS) was not found. For the sake of comparison, we show the energy of the first constrained optimization along the S_1 reaction path.
[b]TIDIR coincides with CoIn.
[c]Not Available due to technical issues in the RS calculation for QuasAr2.

Supplementary Table 5. S_1 energy profile along the MEP calculated at SA2-CASSCF/AMBER level of theory for the wild-type rhodopsins Arch2 and Arch3 and Arch3 engineered mutants QuasAr1, Archon2, QuasAr2, Arch7, Arch5. The paths were calculated as relaxed scans around the C12-C13-C14-C15 dihedral angle (α). The charges shown are Mulliken charges associated to the C14-C15-N moiety. Dipole transition strength for the S_1-S_0 transition is also given (f_{S1-S0}). Shaded in light gray, the rows corresponding to the FC, FS, TS_{S1}, CoIn and TIDIR geometries (see footnotes).

SA2-CASSCF/AMBER						
**α (degres)**	**BLA (Å)**	**S_0 Energy (kcal mol^{-1})**	**S_1 Energy (kcal mol^{-1})**	**S_0 Charge (e)**	**S_1 Charge (e)**	**f_{S1-S0}**
			**Arch2**			
**-162[a]**	0.12	0.00	78.05	0.60	0.28	1.37
**-155**	0.02	22.94	61.48	0.39	0.40	1.49
**-150**	0.03	23.56	61.20	0.39	0.41	1.45
**-145**	0.03	24.43	61.03	0.39	0.44	1.39
**-140**	0.03	25.35	60.74	0.40	0.46	1.32
**-135**	0.03	26.84	60.43	0.40	0.48	1.23
**-130**	0.04	28.15	60.20	0.40	0.51	1.12
**-125**	0.04	29.94	59.27	0.40	0.53	1.02
**-120**	0.05	32.17	58.52	0.39	0.55	0.89
**-115**	0.05	33.80	57.20	0.37	0.58	0.75
**-110**	0.05	36.43	54.97	0.35	0.61	0.53
**-105**	0.06	38.26	52.39	0.32	0.65	0.37
**-99[d]**	0.12	48.62	48.71	0.04	0.94	0.00
			**Arch3**			
**-164[a]**	0.11	0.00	76.40	0.61	0.31	1.46
**-155**	0.03	21.47	59.63	0.40	0.43	1.34
**-150**	0.03	22.26	59.44	0.41	0.45	1.30
**-145**	0.03	23.04	59.24	0.41	0.47	1.24
**-140**	0.03	24.40	58.93	0.41	0.49	1.17
**-135**	0.03	25.72	58.57	0.41	0.51	1.09
**-130**	0.04	27.44	58.07	0.40	0.53	1.00
**-125**	0.04	29.60	57.47	0.40	0.55	0.88
**-120**	0.04	31.65	56.55	0.39	0.58	0.76
**-115**	0.05	33.93	55.41	0.37	0.60	0.63
**-110**	0.05	35.90	53.83	0.35	0.63	0.47
**-105**	0.06	39.00	52.28	0.31	0.68	0.32
**-101[d]**	0.10	47.51	47.53	0.15	0.85	0.00
			**QuasAr1**			
**-165[a]**	0.11	0.00	73.89	0.57	0.28	1.45
**-158[b]**	0.03	19.58	59.05	0.36	0.41	1.48
**-150**	0.03	21.12	59.39	0.35	0.43	1.43
**-145**	0.04	22.19	59.51	0.35	0.45	1.38
**-140**	0.04	23.67	59.64	0.35	0.48	1.32
**-135[c]**	0.04	25.40	59.69	0.34	0.50	1.23
**-130**	0.04	27.28	59.61	0.33	0.53	1.14
**-125**	0.05	29.09	59.35	0.33	0.56	1.03
**-120**	0.05	31.40	58.74	0.31	0.58	0.90
**-115**	0.06	33.59	57.95	0.30	0.62	0.76
**-110**	0.06	35.79	57.05	0.28	0.65	0.61

**-105**	0.07	38.46	55.81	0.25	0.69	0.45
**-100**	0.07	40.66	54.06	0.22	0.73	0.29
**-96[e]**	0.11	47.99	50.18	0.01	0.96	0.00
**-96[d]**	0.13	50.46	50.48	0.23	0.73	0.00
			**Archon2**			
**-165[a]**	0.12	0.00	75.18	0.58	0.28	1.42
**-162[b]**	0.03	23.77	59.27	0.35	0.39	1.41
**-155**	0.03	24.09	59.63	0.36	0.40	1.41
**-150**	0.04	24.17	59.87	0.36	0.42	1.41
**-145**	0.04	25.05	60.49	0.35	0.44	1.40
**-140**	0.04	23.95	60.73	0.35	0.46	1.42
**-135**	0.04	25.85	61.18	0.34	0.49	1.34
**-130**	0.05	28.14	61.72	0.34	0.53	1.24
**-125**	0.05	30.70	61.84	0.33	0.56	1.11
**-120[c]**	0.06	33.18	61.88	0.32	0.59	0.98
**-115**	0.06	36.13	61.77	0.31	0.62	0.83
**-110**	0.07	39.08	61.38	0.29	0.66	0.67
**-105**	0.07	42.21	60.71	0.26	0.70	0.50
**-100**	0.08	45.48	59.93	0.21	0.76	0.32
**-99[e]**	0.11	53.32	55.89	0.04	0.94	0.01
**-99[d]**	0.13	56.08	56.10	0.93	0.05	0.00
			**QuasAr2**			
**-165[a]**	0.11	0.00	72.07	0.54	0.27	1.52
**-153[b]**	0.03	19.01	59.01	0.34	0.40	1.55
**-145**	0.04	20.36	59.15	0.34	0.43	1.48
**-140**	0.04	21.71	59.32	0.33	0.46	1.42
**-135[c]**	0.04	23.35	59.35	0.33	0.49	1.33
**-130**	0.05	25.04	59.31	0.32	0.53	1.23
**-125**	0.05	27.24	59.07	0.31	0.56	1.10
**-120**	0.05	29.35	58.59	0.30	0.60	0.95
**-115**	0.06	31.61	57.81	0.28	0.63	0.80
**-110**	0.06	34.76	56.90	0.26	0.68	0.61
**-105**	0.08	37.02	55.19	0.21	0.74	0.42
**-100**	0.08	39.51	53.84	0.17	0.79	0.26
**-99[e]**	0.11	45.76	51.46	0.01	0.95	0.00
**-98[d]**	0.15	52.51	52.54	0.73	0.22	0.01
			**Arch7**			
**-165[a]**	0.12	0.00	70.79	0.57	0.26	1.59
**-161[b]**	0.03	17.65	55.22	0.33	0.41	1.63
**-155**	0.04	18.44	55.39	0.33	0.43	1.55
**-150**	0.04	19.51	55.80	0.33	0.45	1.49
**-145**	0.04	20.87	56.03	0.32	0.47	1.48
**-140**	0.04	22.26	56.30	0.32	0.50	1.41
**-135**	0.05	23.93	56.51	0.32	0.52	1.31
**-130**	0.05	26.02	56.68	0.31	0.56	1.20
**-125[c]**	0.05	28.18	56.70	0.30	0.59	1.06
**-120**	0.06	30.37	56.50	0.28	0.62	0.92
**-115**	0.06	32.69	56.02	0.26	0.66	0.76
**-110**	0.07	35.18	55.45	0.23	0.70	0.58

**-105**	0.08	38.08	55.02	0.19	0.75	0.40
**-103[e]**	0.10	45.42	52.82	0.02	0.94	0.00
**-102[d]**	0.17	54.22	54.37	0.01	0.94	0.02
			**Arch5**			
**-163[a]**	0.10	0.00	66.38	0.53	0.26	1.55
**-154[b]**	0.04	16.13	53.37	0.30	0.43	1.57
**-145**	0.04	17.31	53.68	0.30	0.46	1.50
**-140**	0.04	18.53	53.98	0.29	0.49	1.44
**-135**	0.04	19.83	54.17	0.29	0.52	1.36
**-130[c]**	0.05	21.64	54.23	0.28	0.55	1.26
**-125**	0.05	23.32	54.17	0.27	0.59	1.14
**-120**	0.06	25.44	54.05	0.26	0.62	1.00
**-115**	0.06	27.34	53.78	0.24	0.66	0.85
**-110**	0.06	29.67	53.21	0.23	0.70	0.68
**-105**	0.07	32.46	52.65	0.20	0.74	0.51
**-100**	0.08	35.48	51.81	0.16	0.79	0.33
**-95[e]**	0.11	43.09	49.28	0.02	0.97	0.00
**-94[d]**	0.16	50.74	50.86	0.97	0.01	0.09

[a] Ground state (S_0) optimized geometry (FC).
[b] planar S_1 minimum (FS) geometry.
[c] S_1 transition state (TS_{S1}) geometry.
[d] Conical Intersection (CoIn) geometry.
[e] Twisted S_1 minimum (TIDIR) geometry.

Supplementary Table 6. S_1 energy profile along the MEP calculated at XMS-CASPT2/AMBER level of theory for the wild-type rhodopsins Arch2 and Arch3 and Arch3 engineered mutants QuasAr1, Archon2, QuasAr2, Arch7, Arch5. Details on how the paths were computed are given in Supplementary Methods 5. XMS-CASPT2 energies for the S_0, S_1 and S_2 states are shown. The Mulliken charges associated to the C14-C15-N moiety, were calculated at the XMCQDPT2/AMBER$_{charges}$ level of theory at the corresponding XMS-CASPT2 corrected geometries. Shaded in light gray, the rows corresponding to the FC, FS, TS_{S1}, and TIDIR geometries (see footnotes).

XMS-CASPT2/AMBER							
**α (degrees)**	**BLA (A)**	**S_0 Energy (kcal mol^{-1})**	**S_1 Energy (kcal mol^{-1})**	**S_2 Energy (kcal mol^{-1})**	**S_0 Charge (e)**	**S_1 Charge (e)**	**S_2 Charge (e)**
**Arch2**							
**-158**[a]	0.06	0.00	52.29	67.88	0.57	0.33	0.64
**-153**[b]	-0.01	17.23	51.23	77.06	0.37	0.43	0.66
**-149**	0.00	16.72	51.12	76.75	0.37	0.44	0.68
**-139**[c]	0.01	17.59	51.33	78.57	0.35	0.50	0.71
**-128**	0.01	19.04	51.18	81.35	0.28	0.59	0.75
**-118**	0.01	19.78	51.19	85.02	0.19	0.71	0.81
**-108**	0.02	20.64	50.15	89.18	0.07	0.82	0.88
**-97**[d]	0.08	24.59	48.01	90.37	-0.02	0.93	0.93
**Arch3**							
**-162**[a]	0.06	0.00	52.71	67.43	0.55	0.34	0.64
**-154**[b]	0.00	16.35	50.51	76.34	0.37	0.46	0.67
**-149**	0.00	16.70	50.58	77.16	0.35	0.49	0.69
**-139**	0.01	17.69	50.87	79.35	0.32	0.56	0.73
**-129**[c]	0.01	18.67	51.00	82.29	0.26	0.65	0.78
**-119**	0.02	19.95	50.79	85.94	0.18	0.75	0.85
**-108**	0.02	20.64	49.67	88.48	0.08	0.84	0.89
**-99**[d]	0.08	25.20	47.13	88.61	-0.01	0.94	0.94
**QuasAr1**							
**-161**[a]	0.05	0.00	51.32	65.84	0.54	0.32	0.59
**-156**[b]	0.01	14.97	50.00	74.29	0.35	0.43	0.64
**-148**	0.01	15.98	50.56	75.84	0.35	0.46	0.66
**-138**	0.02	17.56	51.39	78.42	0.31	0.53	0.70
**-128**	0.02	19.76	52.15	82.27	0.25	0.63	0.74
**-118**[c]	0.03	20.38	52.38	85.61	0.19	0.73	0.81
**-108**	0.04	21.57	52.34	89.43	0.11	0.82	0.88
**-94**[d]	0.08	26.13	49.57	90.68	0.00	0.95	0.95
**Archon2**							
**-164**[a]	0.05	0.00	51.08	65.74	0.55	0.33	0.59
**-162**[b]	0.00	17.92	50.29	78.10	0.36	0.44	0.64
**-149**	0.01	18.20	51.28	78.76	0.35	0.46	0.66
**-139**	0.01	17.62	52.14	78.34	0.33	0.50	0.68
**-129**	0.02	20.03	53.89	81.84	0.28	0.59	0.71
**-119**	0.01	23.34	55.53	85.97	0.23	0.66	0.74
**-109**	0.03	24.25	56.68	92.63	0.11	0.73	0.78
**-98**[c, d]	0.08	33.13	56.71	98.24	-0.03	0.94	0.94

			**QuasAr2**				
**-161**[a]	0.05	0.00	50.61	65.28	0.52	0.32	0.57
**-152**	0.02	14.21	50.55	73.38	0.36	0.43	0.64
**-139**	0.02	16.29	51.21	76.89	0.32	0.50	0.68
**-128**	0.02	18.51	52.02	80.32	0.25	0.60	0.71
**-118**	0.03	19.69	52.57	84.23	0.17	0.72	0.78
**-108**	0.04	20.98	52.95	89.63	0.08	0.83	0.86
**-100**[d]	0.08	22.97	47.61	88.48	-0.02	0.93	0.94
			**Arch7**				
**-163**[a]	0.05	0.00	48.92	64.18	0.49	0.31	0.58
**-160**[b]	0.01	12.25	48.56	73.71	0.32	0.45	0.65
**-149**	0.02	13.77	49.91	76.27	0.30	0.49	0.68
**-139**	0.02	15.40	51.08	79.05	0.27	0.56	0.70
**-129**	0.03	17.58	52.65	82.75	0.21	0.65	0.73
**-119**	0.04	19.65	54.24	87.53	0.13	0.75	0.79
**-109**[c]	0.04	21.05	54.88	92.41	0.05	0.85	0.86
**-102**[d]	0.07	25.55	54.83	96.54	-0.03	0.93	0.93
			**Arch5**				
**-161**[a]	0.05	0.00	48.22	63.04	0.46	0.29	0.51
**-153**[b]	0.01	13.24	47.16	74.39	0.27	0.46	0.61
**-139**	0.02	14.10	48.42	75.93	0.26	0.52	0.66
**-129**	0.02	16.00	48.96	78.31	0.22	0.60	0.69
**-119**	0.03	17.21	49.97	81.44	0.15	0.66	0.74
**-109**[c]	0.04	17.22	50.89	87.19	0.08	0.77	0.80
**-94**[d]	0.06	20.78	49.93	91.28	-0.01	0.94	0.96

[a]Ground state (S_0) geometry (FC).
[b]Fluorescent state (FS) geometry.
[c]S_1 transition state (TS_{S1}) geometry.
[d]Twisted S_1 minimum (TIDIR) geometry.

Supplementary Table 7. Mayer Analysis of free valences (number of unpaired electrons) based on the S_1 XMCQDPT2/AMBER densities for the QM/MM models of Arch3 and Arch7 at FC, FS and TIDIR geometries.

**Number of unpaired electrons (NUE)**			
**Arch3**			
**Atom**	**FC**	**FS**	**TIDIR**
C5	0.209	0.218	0.256
C6	0.158	0.156	0.173
C7	0.236	0.240	0.361
C8	0.186	0.170	0.211
C9	0.245	0.223	0.393
C10	0.281	0.196	0.206
C11	0.361	0.225	0.345
C12	0.363	0.222	0.175
C13	0.437	0.205	0.262
C14	0.329	0.278	0.597
C15	0.293	0.165	0.206
N	0.165	0.166	0.333
**Arch7**			
C5	0.216	0.219	0.250
C6	0.160	0.159	0.173
C7	0.224	0.253	0.361
C8	0.205	0.186	0.207
C9	0.258	0.253	0.395
C10	0.321	0.239	0.206
C11	0.382	0.258	0.336
C12	0.371	0.269	0.175
C13	0.412	0.225	0.251
C14	0.285	0.266	0.609
C15	0.253	0.166	0.201
N	0.167	0.184	0.355

Supplementary Table 8. Mayer analysis of the bond order based on S_1 XMCQDPT2/AMBER densities for the QM/MM models of Arch3 and Arch7 at FC, FS and TIDIR geometries.

Bond Order Analysis			
**Arch3**			
**Bond**	**FC**	**FS**	**TIDIR**
N-C15	1.271	1.278	1.418
C14-C15	1.392	1.432	1.237
C13-C14	1.215	1.141	1.071
C12-C13	1.296	1.530	1.590
C11-C12	1.297	1.111	1.145
C10-C11	1.290	1.578	1.452
C9-C10	1.333	1.151	1.273
C8-C9	1.296	1.441	1.297
C7-C8	1.411	1.269	1.430
C6-C7	1.240	1.288	1.181
C5-C6	1.528	1.475	1.595
**Arch7**			
N-C15	1.244	1.236	1.416
C14-C15	1.383	1.477	1.256
C13-C14	1.212	1.083	1.045
C12-C13	1.276	1.598	1.612
C11-C12	1.311	1.087	1.131
C10-C11	1.274	1.602	1.445
C9-C10	1.353	1.153	1.273
C8-C9	1.260	1.427	1.304
C7-C8	1.458	1.300	1.430
C6-C7	1.189	1.253	1.174
C5-C6	1.558	1.487	1.580

Supplementary Table 9. XMS-CASPT2/AMBER S_1 energy profile along the MEP for 3 representative models (Arch3, QuasAr1 and Arch7) calculated *in vacuum* (i.e. in absence of the protein environment). The chromophore geometries are supposed the same as in the corresponding XMS-CASPT2/AMBER reaction paths calculated in the presence of the protein.

α (degrees)	BLA (Å)	S_0 Energy (kcal mol^{-1})	S_1 Energy (kcal mol^{-1})	S_2 Energy (kcal mol^{-1})	$\Delta E^{RS\text{-}FS}_{S1}$ (kcal mol^{-1})
			**Arch3**		
**-162**	0.06	0.0	47.7	65.2	0.0
**-154**	0.00	12.6	47.9	74.9	0.3
**-149**	0.00	13.4	48.6	76.0	0.9
**-139**	0.01	14.8	51.0	79.2	3.4
**-129**	0.01	16.7	52.0	79.7	4.4
**-119**	0.02	19.3	52.2	78.4	4.5
**-108**	0.02	22.7	48.3	76.7	0.6
**-99**	0.08	26.3	46.4	77.5	-1.2
			**QuasAr1**		
**-161**	0.05	0.0	47.2	64.2	0.0
**-156**	0.01	11.4	47.9	75.0	0.7
**-148**	0.01	12.4	48.7	76.3	1.4
**-138**	0.02	14.3	50.0	78.3	2.8
**-128**	0.02	16.7	50.6	80.2	3.3
**-118**	0.03	18.8	52.4	81.6	5.2
**-108**	0.04	22.5	52.0	79.3	4.7
**-98**	0.05	26.1	50.3	79.4	3.1
**-94**	0.08	28.8	47.5	79.7	0.3
			**Arch7**		
**-163**	0.05	0.0	46.5	62.2	0.0
**-160**	0.01	10.8	47.0	72.0	0.5
**-149**	0.02	12.1	48.2	74.2	1.7
**-139**	0.02	13.8	49.6	76.8	3.2
**-129**	0.03	15.8	52.1	79.6	5.6
**-119**	0.04	18.3	52.6	79.1	6.2
**-109**	0.04	21.6	51.6	77.6	5.1
**-102**	0.07	26.1	45.2	77.4	-1.3

[a]**$\Delta E^{RS\text{-}FS}_{S1}$** corresponds to the energy difference between the reaction path at each point and the S_1 Energy at the first point shown for each model.

Supplementary Table 10. XMS-CASPT2/AMBER S_1 energy profile along the MEP of 3 representative models (Arch3, QuasAr1 and Arch7) calculated in the presence of the opsins van der Waals interaction only (the MM electrostatics contribution of the protein is neglected). The geometries were kept frozen at the same positions as in the corresponding XMS-CASPT2/AMBER reaction paths calculated with a full QMMM scheme.

**α (degrees)**	**BLA (Å)**	**S_0 Energy (kcal mol^{-1})**	**S_1 Energy (kcal mol^{-1})**	**S_2 Energy (kcal mol^{-1})**	**$^{(a)}\Delta E^{RS\text{-}FS}_{S1}$ (kcal mol^{-1})**
		**Arch3**			
**-162**	0.06	0.0	46.3	63.8	
**-154**	0.00	5.9	41.2	68.2	0.0
**-149**	0.00	6.5	41.7	69.0	0.4
**-139**	0.01	7.6	43.8	71.9	2.5
**-129**	0.01	9.0	44.4	72.1	3.2
**-119**	0.02	12.0	42.5	70.5	1.2
**-108**	0.02	15.0	40.6	69.0	-0.7
**-99**	0.08	18.8	39.0	70.1	-2.3
		**QuasAr1**			
**-161**	0.05	0.0	46.0	63.0	
**-156**	0.01	10.2	46.3	71.9	0.0
**-148**	0.01	11.2	47.2	73.3	0.9
**-138**	0.02	13.1	48.9	75.5	2.5
**-128**	0.02	16.3	50.3	76.7	4.0
**-118**	0.03	18.7	50.8	76.5	4.5
**-108**	0.04	21.5	50.9	76.6	4.6
**-98**	0.05	24.7	50.7	77.5	4.3
**-94**	0.08	27.0	50.9	78.0	4.6
		**Arch7**			
**-163**	0.05	0.0	46.5	62.2	
**-160**	0.01	9.7	45.8	70.8	0.0
**-149**	0.02	10.4	46.5	72.5	0.7
**-139**	0.02	11.6	47.4	74.5	1.6
**-129**	0.03	12.7	49.0	76.6	3.2
**-119**	0.04	14.6	48.9	75.4	3.1
**-109**	0.04	17.4	47.5	73.4	1.6
**-102**	0.07	23.1	42.2	74.3	-3.7

$^{a}\Delta E^{RS\text{-}FS}_{S1}$ corresponds to the energy difference between the reaction path at each point and the S_1 Energy at the first point shown for each model.

Supplementary Table 11. S_1 SA2-CASSCF/AMBER S_1 energy profile along the MEP re-calculated in the presence of the opsins van der Waals interaction only (the MM electrostatics contribution of the protein is neglected). The torsional angle α is constrained at the value used in the original reaction path (i.e., the QMMM path calculated at the same level of theory). The energies given are relative to the S_0 Energy of the FS geometry (first row) for each model (Arch3, QuasAr1, Arch7).

**α (degrees)**	**BLA (Å)**	**S_0 Energy (kcal mol^{-1})**	**S_1 Energy (kcal mol^{-1})**	**$^{(a)}\Delta E^{RS\text{-}FS}_{S1}$ (kcal mol^{-1})**
		**Arch3**		
**-155**	0.05	0.0	38.3	0.0
**-150**	0.05	1.2	39.0	0.7
**-145**	0.05	2.8	39.9	1.6
**-140**	0.06	4.5	40.7	2.4
**-135**	0.06	6.5	41.5	3.3
**-125**	0.07	11.0	42.6	4.3
**-120**	0.08	13.2	42.9	4.6
**-115**	0.08	15.4	42.9	4.6
**-110**	0.09	17.2	42.8	4.5
**-105**	0.09	19.6	42.4	4.1
**-101**	0.11	23.2	41.1	2.8
		**QuasAr1**		
**-158**	0.05	0.0	38.5	0.0
**-150**	0.05	1.6	39.6	1.1
**-145**	0.05	3.0	40.3	1.9
**-140**	0.06	4.7	41.2	2.7
**-135**	0.06	6.6	42.0	3.5
**-130**	0.07	8.6	42.6	4.2
**-125**	0.07	10.1	42.7	4.2
**-120**	0.08	12.0	42.8	4.3
**-115**	0.08	14.0	42.6	4.1
**-110**	0.09	15.7	42.3	3.8
**-105**	0.09	18.2	42.3	3.8
**-100**	0.10	19.9	41.5	3.0
**-96**	0.11	22.3	38.5	0.0
**-91**	0.11	21.5	38.2	-0.3
		**Arch7**		
**-158**	0.04	0.0	39.1	0.0
**-155**	0.05	0.6	39.4	0.2
**-150**	0.05	1.4	39.8	0.7
**-145**	0.05	2.4	40.4	1.2
**-140**	0.05	3.7	41.0	1.8
**-135**	0.06	5.2	41.6	2.5
**-130**	0.06	7.0	42.2	3.1
**-125**	0.07	9.1	42.6	3.5
**-120**	0.08	11.3	42.9	3.7
**-115**	0.08	13.5	42.9	3.8
**-110**	0.09	15.7	42.8	3.6
**-105**	0.09	17.8	42.5	3.4
**-97**	0.11	22.4	41.1	2.0

$^{a}\Delta E^{RS\text{-}FS}_{S1}$ corresponds to the energy difference between the reaction path at each point and the S_1 Energy at the first point shown for each model.

Supplementary Figures

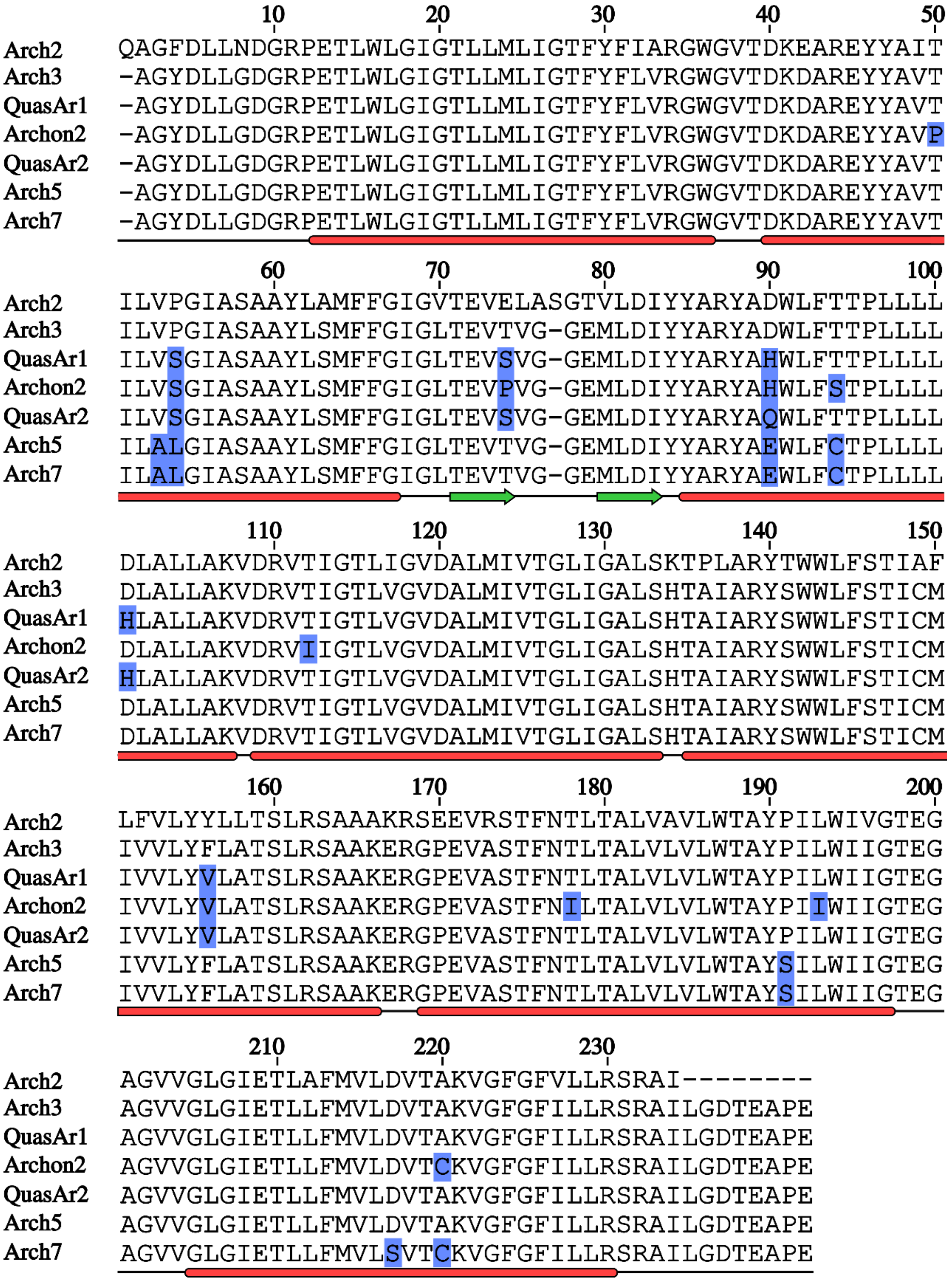

Supplementary Figure 1. **Arch-set protein sequences.** Amino acid sequences alignment performed by ClustalW of the models studied in this work. Arch2 and Arch3 are wild type rhodopsins, while QuasAr1, QuasAr2, Arch5 and Arch7 are Arch3 mutants. Parts in blue corresponds to sequence differences between Arch2 and the other models, while parts in green and red show Arch3 to QuasAr1 or QuasAr2 mutations and Arch3 to Arch5 or Arch7 mutations, respectively.

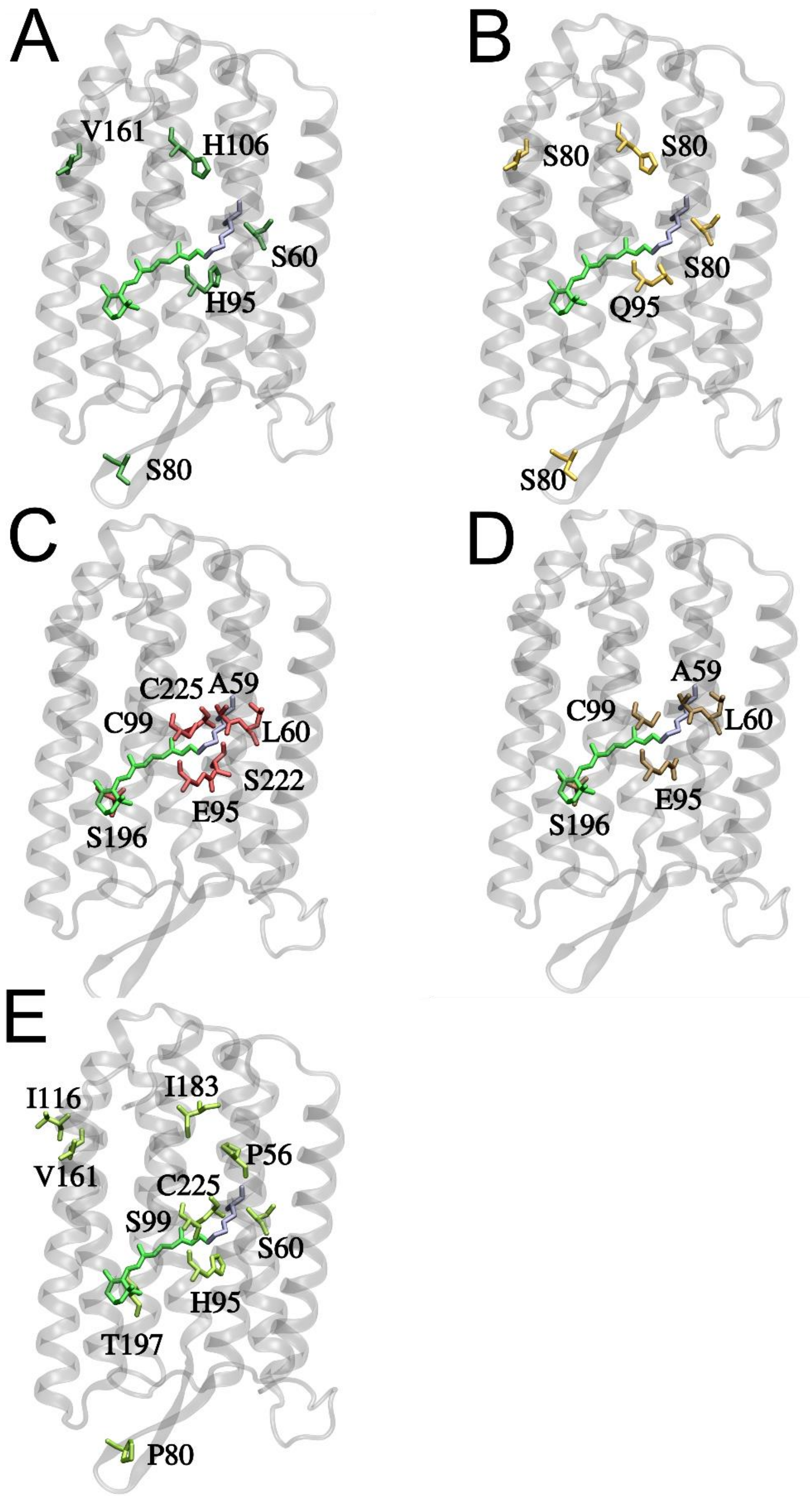

Supplementary Figure 2. **Arch3-mutants side-chains substitutions.** Pictorial representation of the mutations localization in QuasAr1 (A), QuasAr2 (B), Arch7 (C) and Arch5 (D) with respect to the parent wild-type rhodopsin Arch3. Mutated amino acids are shown in sticks and the corresponding label is given. The retinal and linker-lysine, green and blue ball and sticks, respectively, are also shown.

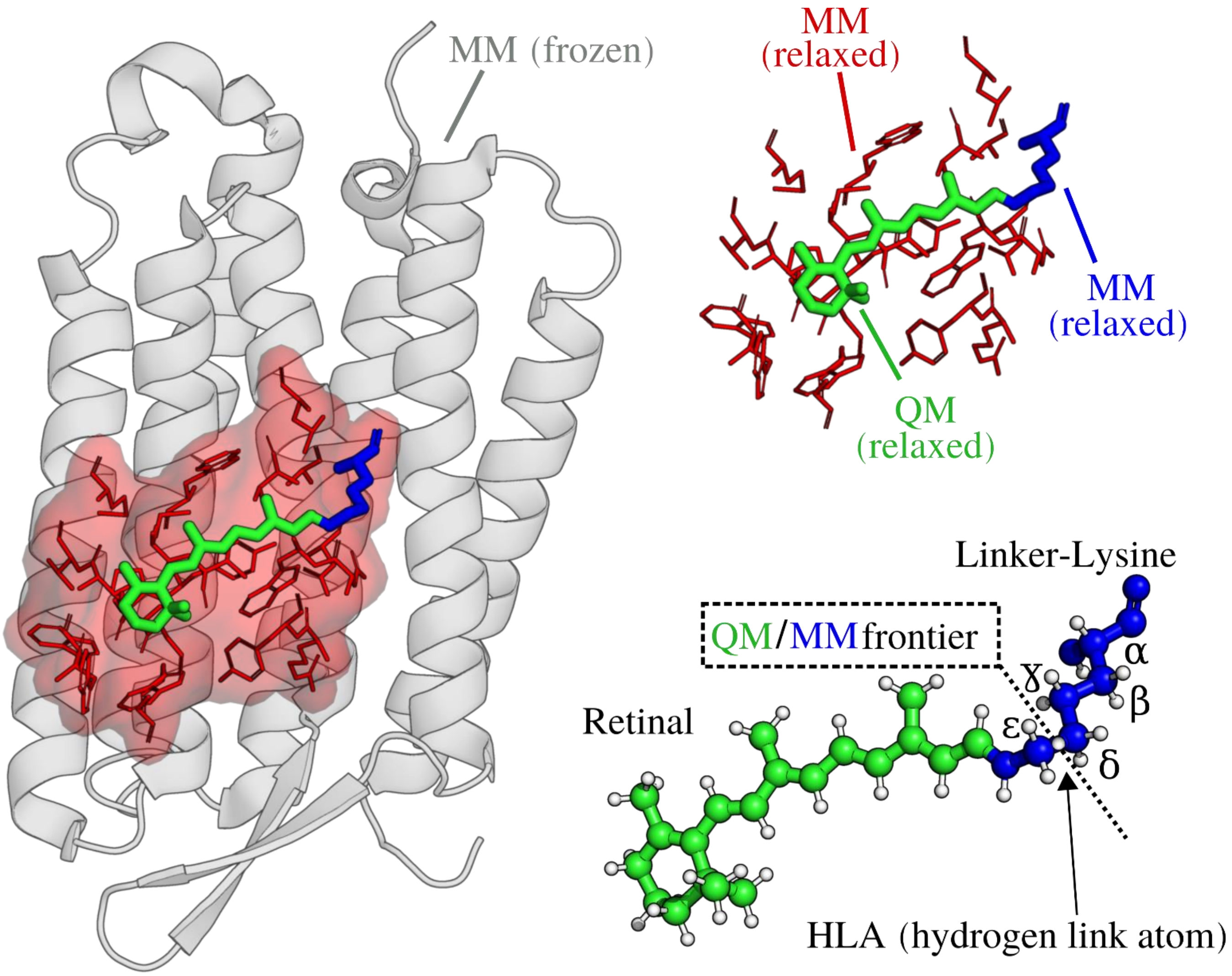

Supplementary Figure 3. The a-ARM protocol QM/MM scheme. Schematic representation of an *in silico* designed QM/MM model generated with *a*-ARM. The *a*-ARM protocol partitions the rhodopsin rhodopsins structures in 3 subsystems which are treated with different approximation along the program workflow. These are the MM frozen atoms (in gray), MM relaxed atoms (in red) and QM atoms (in red). A frontier is defined along the Cδ and Cε carbons of the chromophore linker-Lysine. The QM valence is saturated by a dummy atom called hydrogen link atom (HLA).

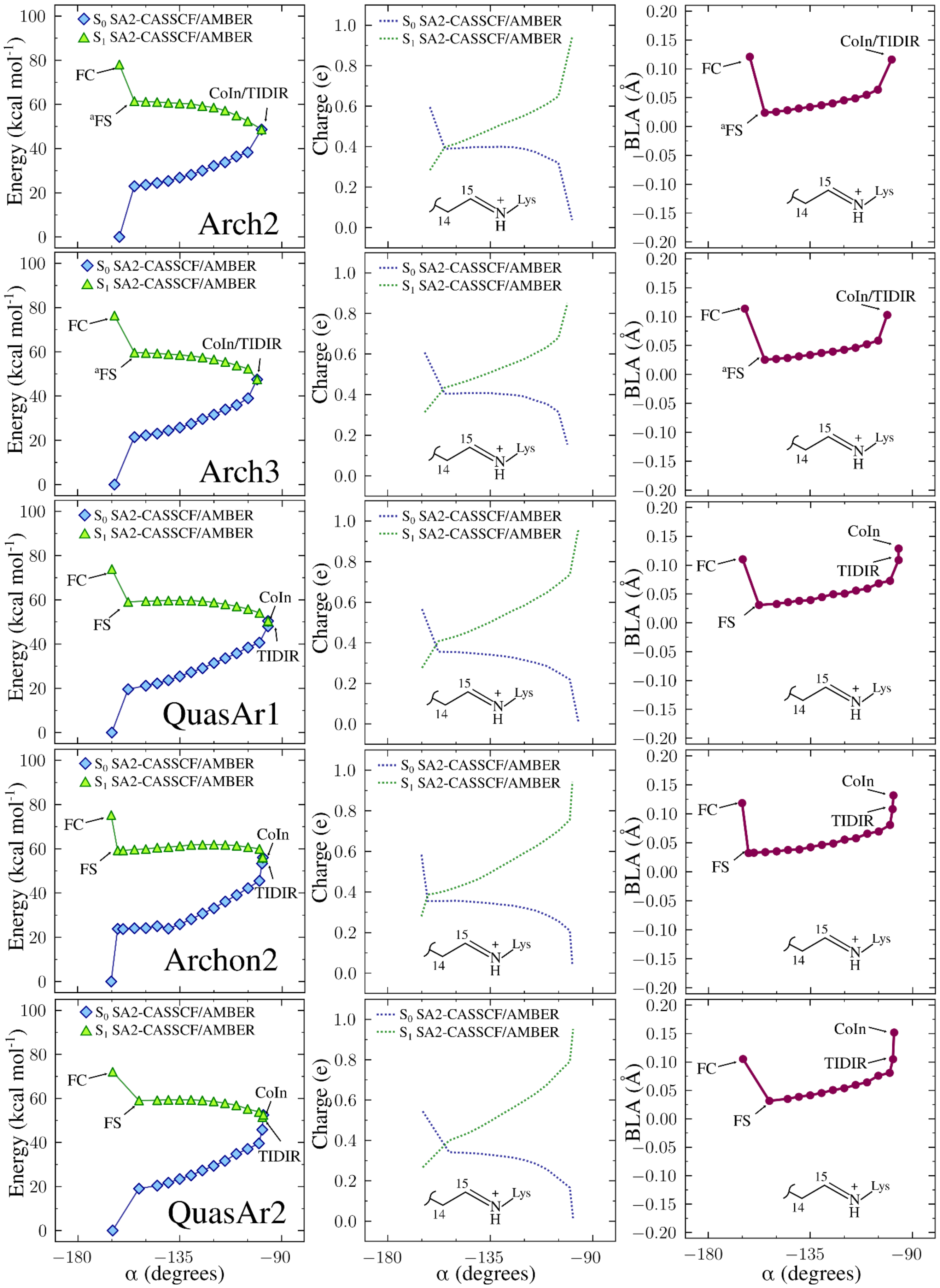

S_0 SA2-CASSCF/AMBER
S_1 SA2-CASSCF/AMBER
FC
^{a}FS
FS
CoIn/TIDIR
CoIn
TIDIR
Arch2
Arch3
QuasAr1
Archon2
QuasAr2
Energy (kcal mol^{-1})
Charge (e)
BLA (Å)
α (degrees)
15
14
Lys
N
H

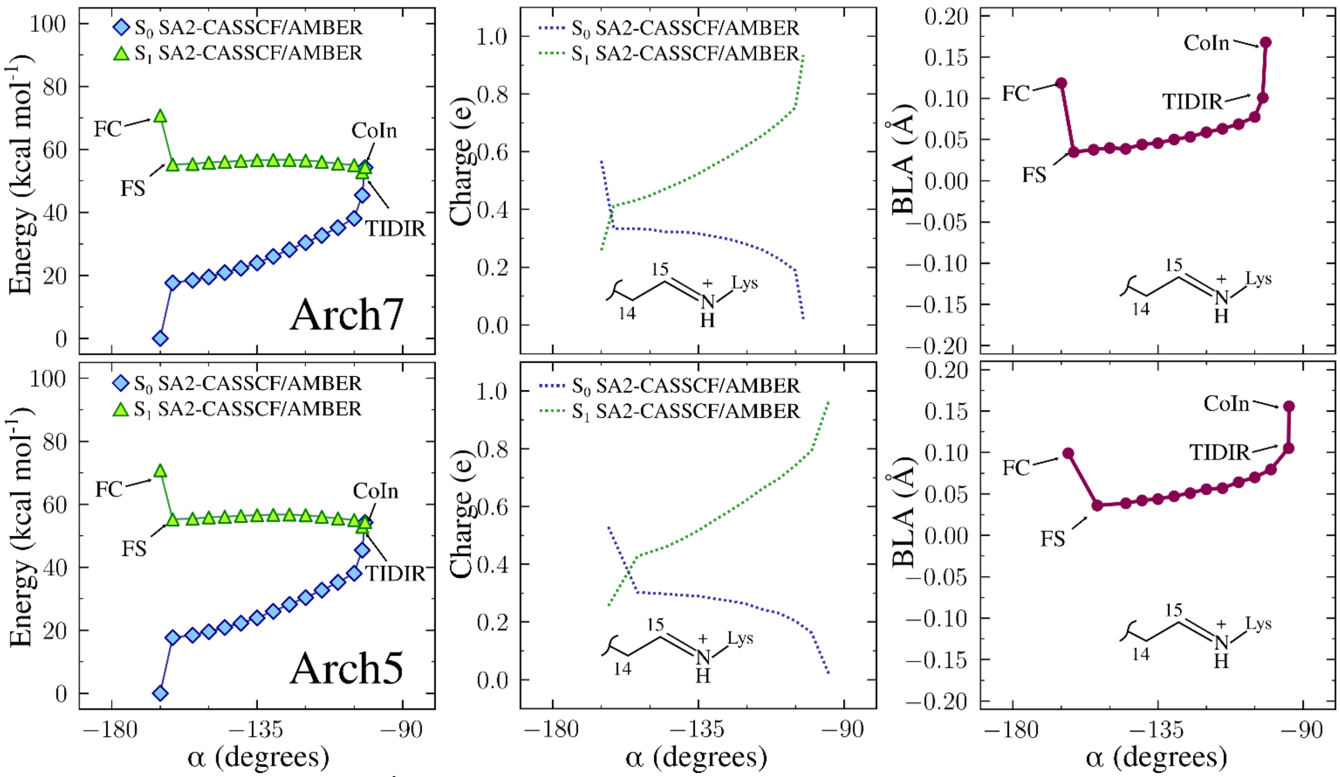

Supplementary Figure 4. 0th order energy profiles along the isomerization coordinate. Overview of energy data from the S_1 QM/MM relaxed scans along α (C12-C13-C14-C15 torsion) calculated at the SA2-CASSCF/AMBER level, for all the rhodopsins of the set. The rows correspond to the models. The columns show: (i) from left to right, energy profiles relative to the S_0 energy of FC (ii) corresponding Mulliken charge of the displayed Schiff base moiety, (iii) change in the BLA geometrical value.

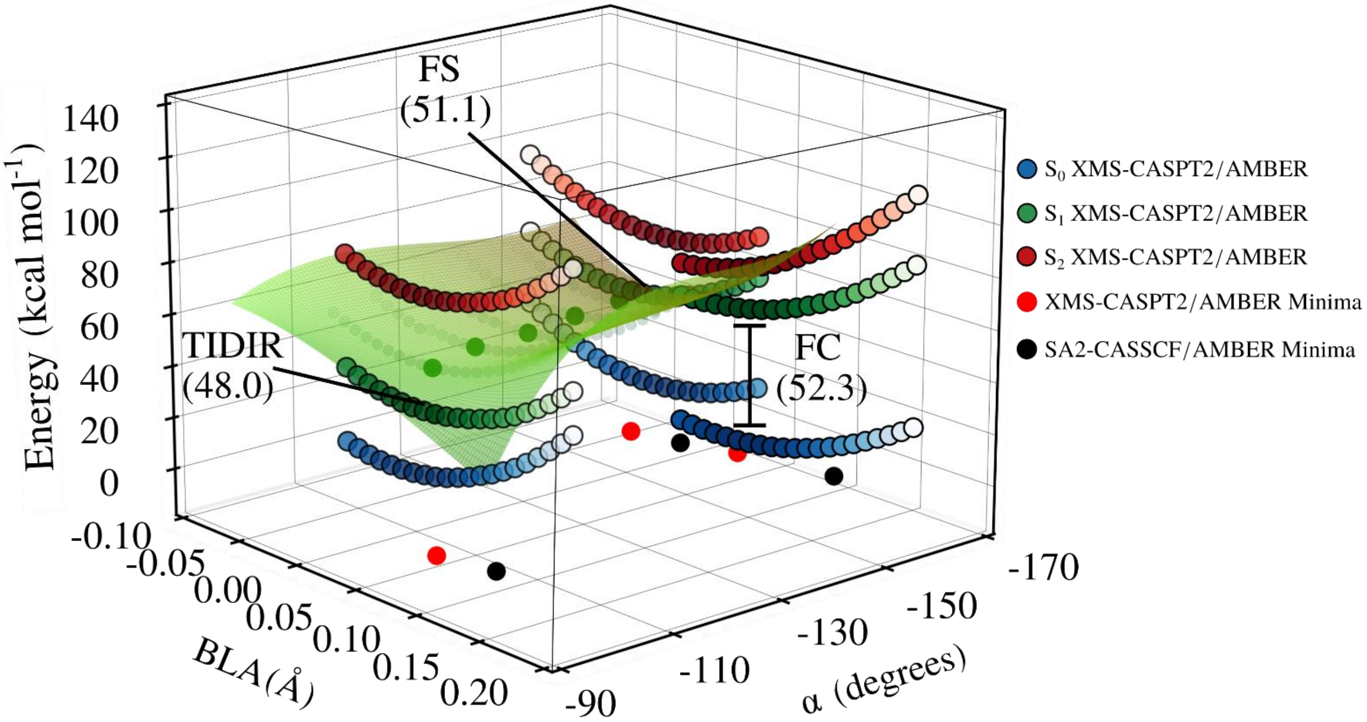

Supplementary Figure 5. Perturbative correction to the 0th order wavefunction. to Schematic representation of the strategy employed to obtain an XMS-CASPT2/AMBER geometrical correction of the SA2-CASSCF/AMBER geometries. The geometries were displayed along a "correction vector" and the energies re-calculated at the XMS-CASPT2/AMBER level. The set of minima located in each cross-section defines the corrected reaction path The positions of the SA2-CASSCF/AMBER relevant FC, FS and TIDIR points and their new corrected XMS-CASPT2/AMBER positions are displayed as colored black and red dots, respectively, on the α, BLA plane. The energies reported are relative to the S_0 energy of the FC structure.

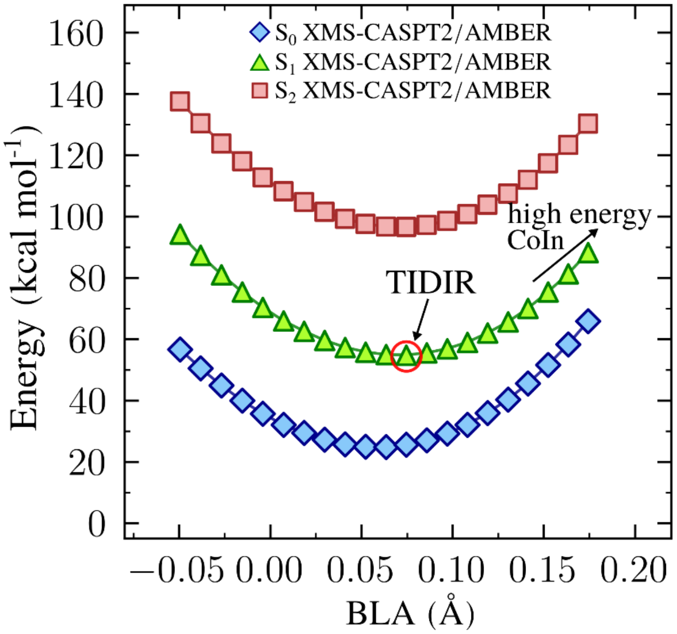

Supplementary Figure 6. CoIn topology after perturbative correction. XMS-CASPT2/AMBER cross-section along the BLA coordinate of the QM/MM model of Arch7 at twisted (ca. 100°) α torsion. The cross-section shows how the XMS-CASPT2/AMBER correction to the SA2-CASSCF/AMBER profiles determine the CoIn topology to be sloped. The energies reported are relative to the S_0 energy of the XMS-CASPT2/AMBER FC structure.

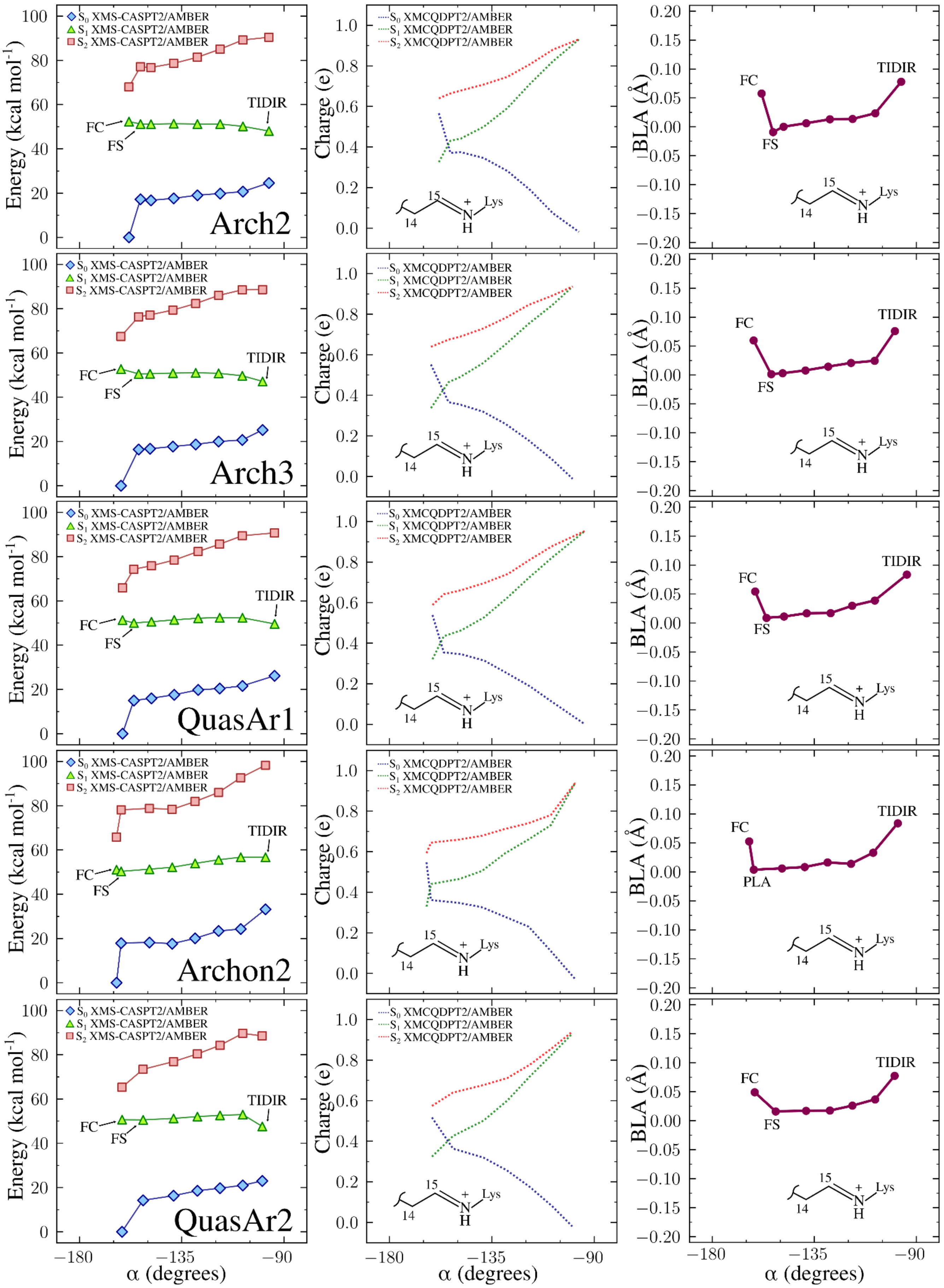
S0 XMS-CASPT2/AMBER
S1 XMS-CASPT2/AMBER
S2 XMS-CASPT2/AMBER
S0 XMCQDPT2/AMBER
S1 XMCQDPT2/AMBER
S2 XMCQDPT2/AMBER
Energy (kcal mol-1)
Charge (e)
BLA (Å)
α (degrees)
FC
FS
PLA
TIDIR
Arch2
Arch3
QuasAr1
Archon2
QuasAr2
14
15
N
H
Lys

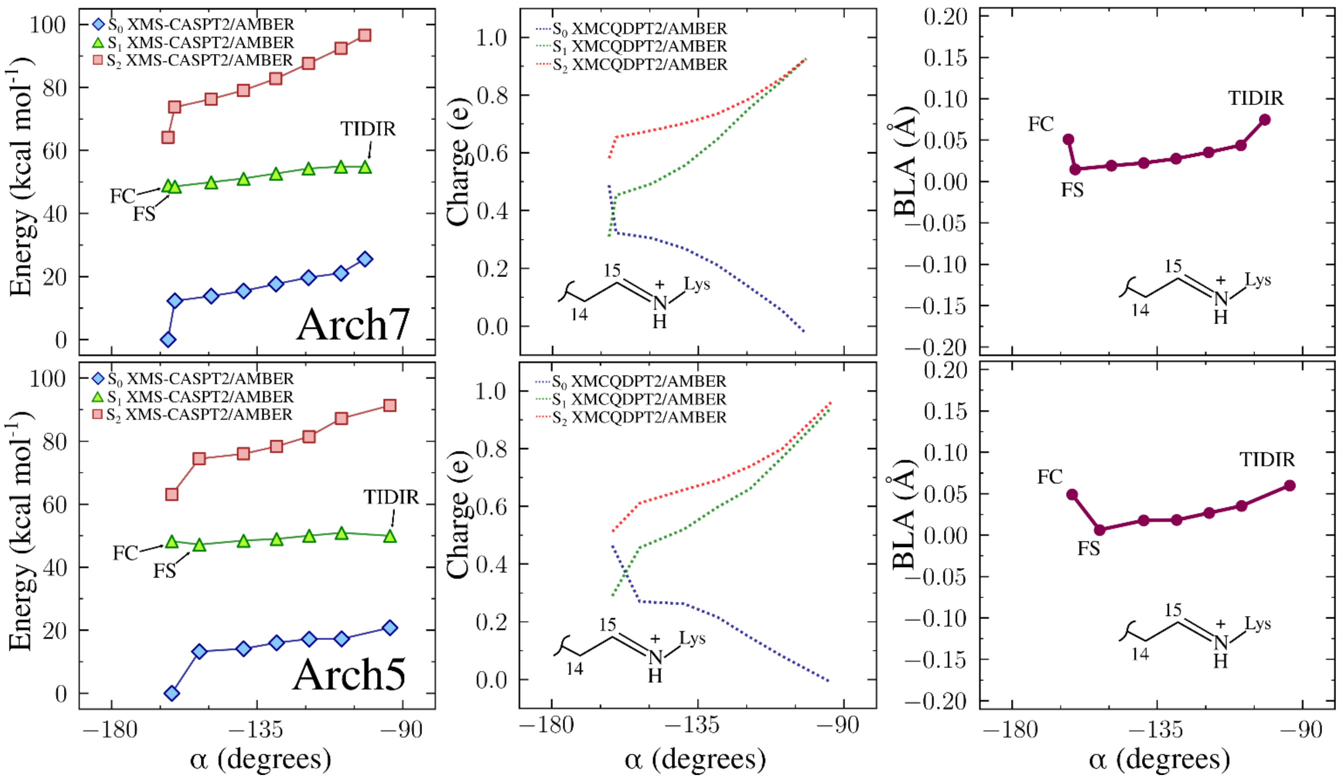

Supplementary Figure 7. **XMS-CASPT2/AMBER energy profiles along the isomerization coordinate** Overview of energy data from the S_1 QM/MM relaxed scans along α (C12-C13-C14-C15 dihedral) calculated at the XMS-CASPT2/AMBER level, for all the rhodopsins of the set. The rows correspond to the models. The columns show: (i) from left to right, energy profiles relative to the S_0 energy of FC, (ii) corresponding Mulliken charge of the displayed Schiff base moiety, (iii) change in the BLA geometrical value.

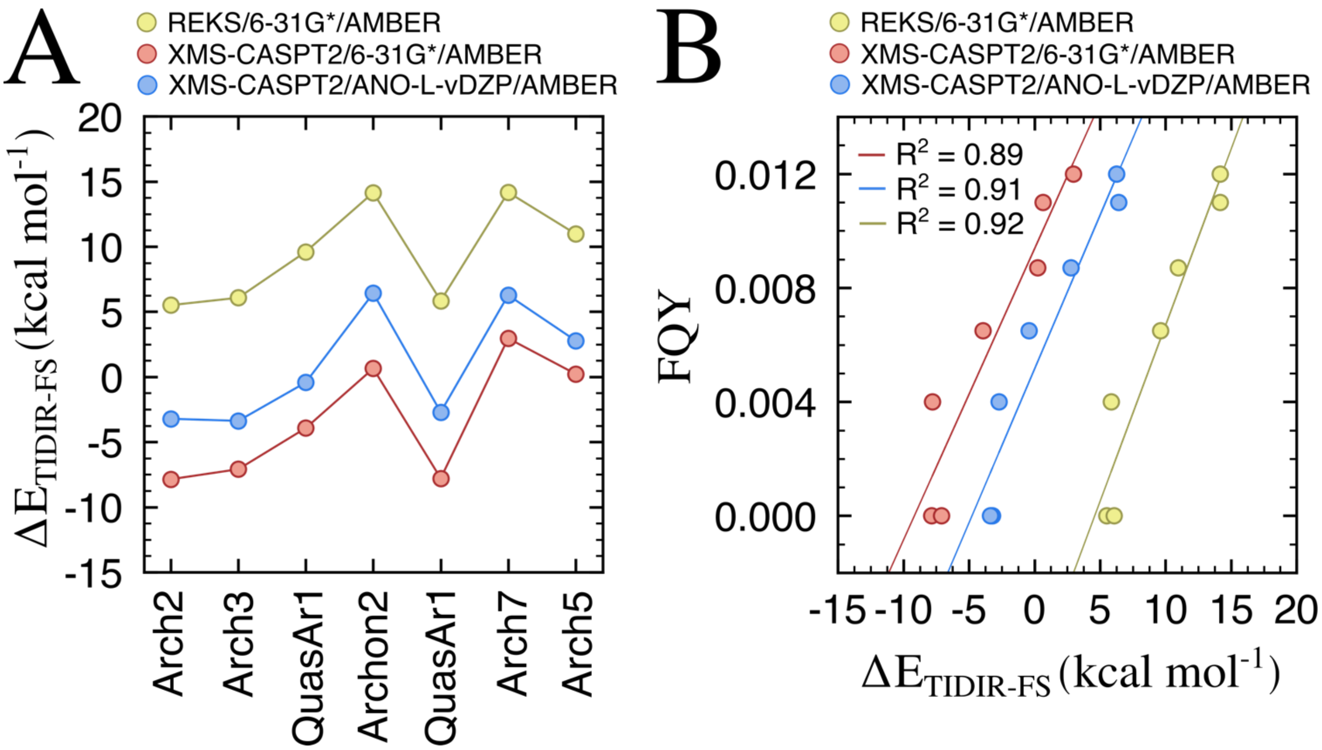

Supplementary Figure 8. SI-SA2-REKS(2,2) calculations of the isomerization energy. A. Dependency of the S_1 reaction energy ($\Delta E_{TIDIR-FS}$) on the level of theory of the QM/MM calculation. The blue curve is obtained from geometric interpolations (see Supplementary text S5) at the SA3-CASSCF(12,12)/XMS-CASPT2/ANO-L-vDZP/AMBER level of theory, the yellow curve from excited state geometry optimization at the SI-SA-REKS(2,2)/6-31G*/AMBER level and finally the red curve from energy correction at the SA2-CASSCF(12,12)/XMS-CASPT2/6-31G*/AMBER level of theory of the SI-SA-REKS(2,2)/6-31G*/AMBER optimized geometries . B. $\Delta E_{TIDIR-FS}$ calculated with different strategies holds the same linear relationship with experimental FQY.

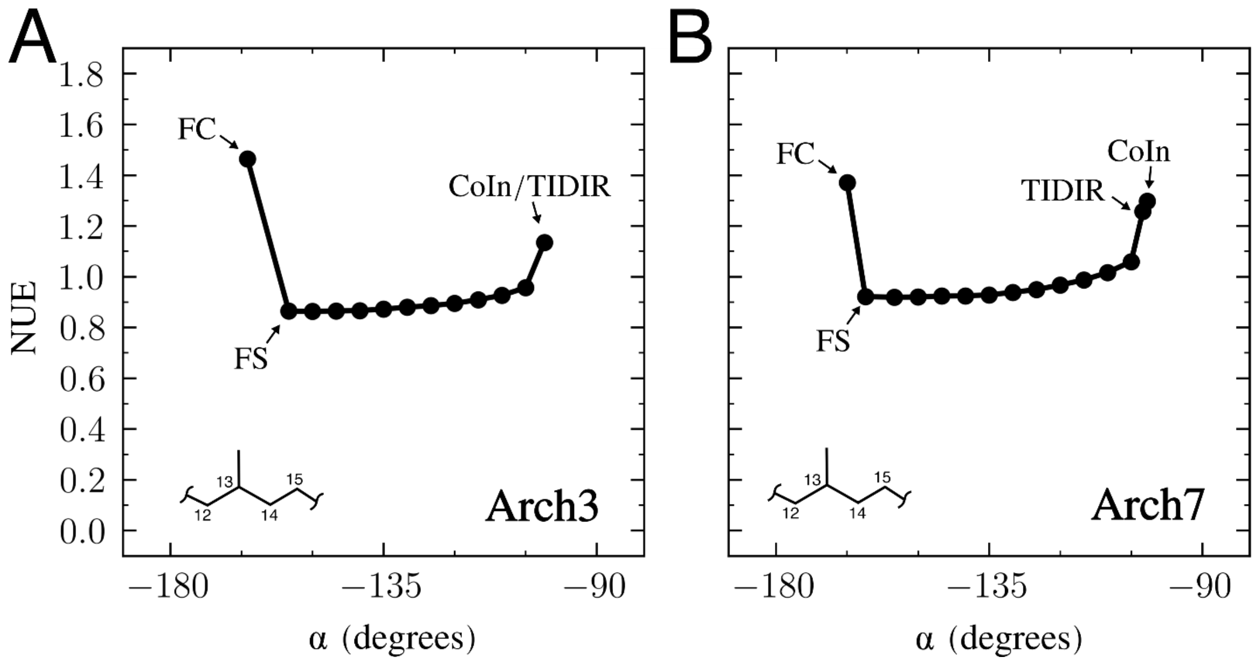

Supplementary Figure 9. Free valence analysis at the 0th order level. Number of unpaired electrons (NUE) on the atoms C12-C13-C14-C15 along the SA2-CASSCF/AMBER reaction paths for Arch3 (left panel) and Arch7 (right panel).

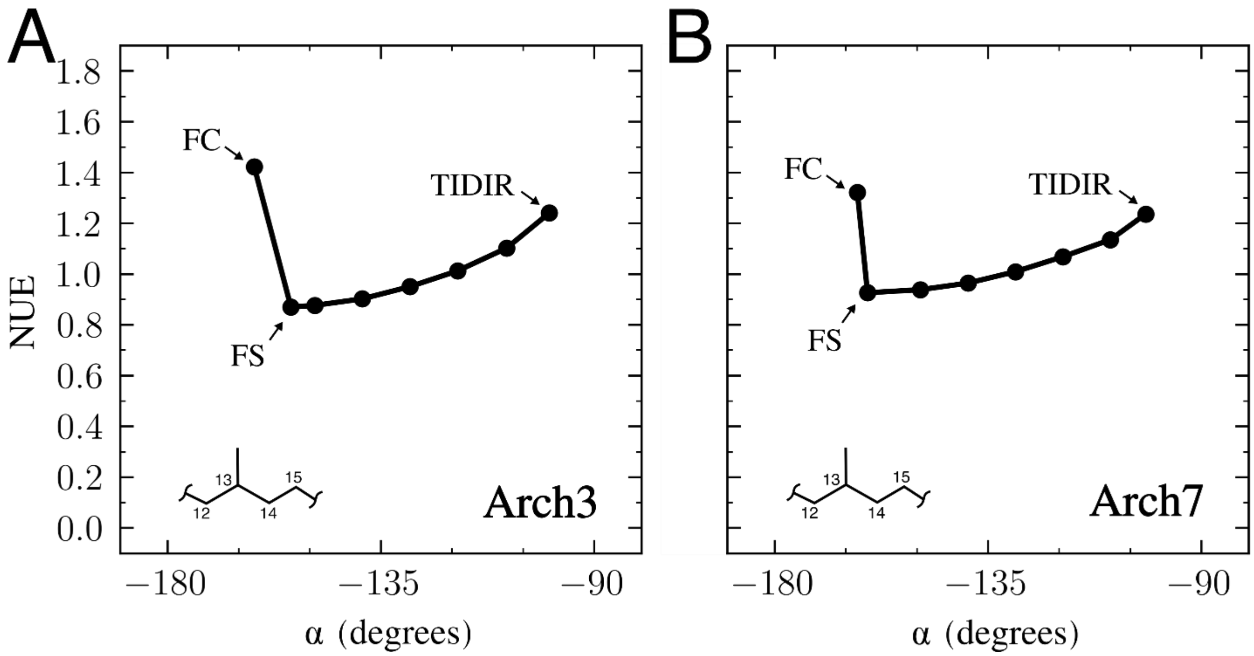

Supplementary Figure 10. Free valence analysis at the 1st order level. Number of unpaired electrons (NUE) on the atoms C12-C13-C14-C15 along the XMS-CASPT2/AMBER reaction paths for Arch3 (left panel) and Arch7 (right panel), using QDPT densities.

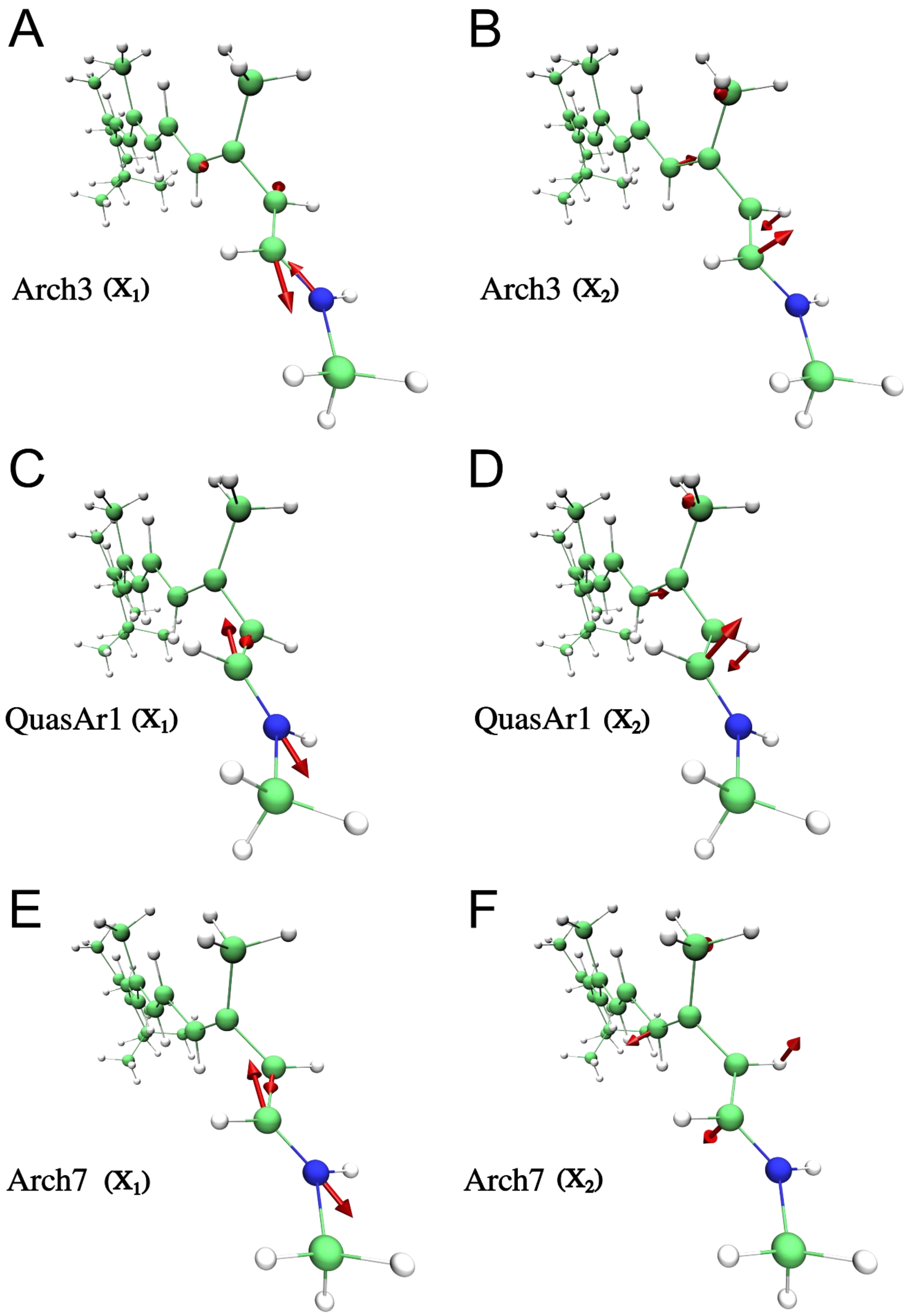

Supplementary Figure 11. Conical Intersections branching plane. (A, B) pictorial representation of the $\boldsymbol{X_1}$ (left) and $\boldsymbol{X_2}$ (right) branching vectors at the chromophore CoIn geometries of Arch3, QuasAr1 (C, D) and Arch7 (E, F).

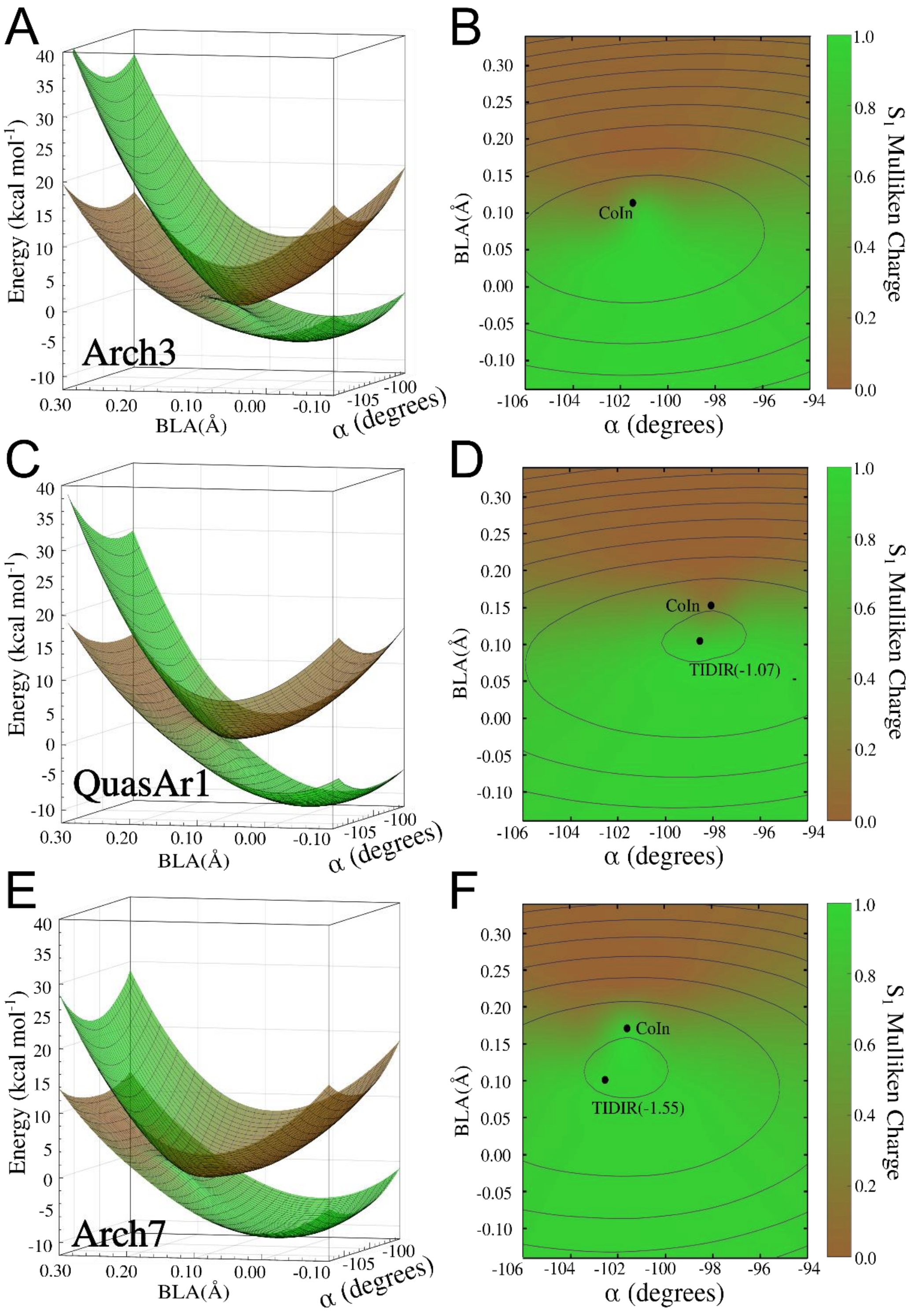

Supplementary Figure 12. **Conical Intersections topologies along the Arch set.** (A) Three-dimensional representation of the SA2-CASSCF/AMBER S_1/S_0 CoIn for Arch3 and corresponding S_1 state contour plot (B). The energies shown are relative to the crossing point energy. The same representations are shown for QuasAr2 (C, D) and Arch7 (E, F). The maps were calculated by sampling the S_0 and S_1 energies at the points of a square 2D grid generated by the $\boldsymbol{X_1}$ and $\boldsymbol{X_2}$ branching vectors of the CoIn. The diagrams highlight the presence, in QuasAr1 (D) and Arch7 (F), of a TIDIR on the excited state close to the surfaces crossing. Conversely in Arch3, TIDIR coincides with the CoIn geometry.

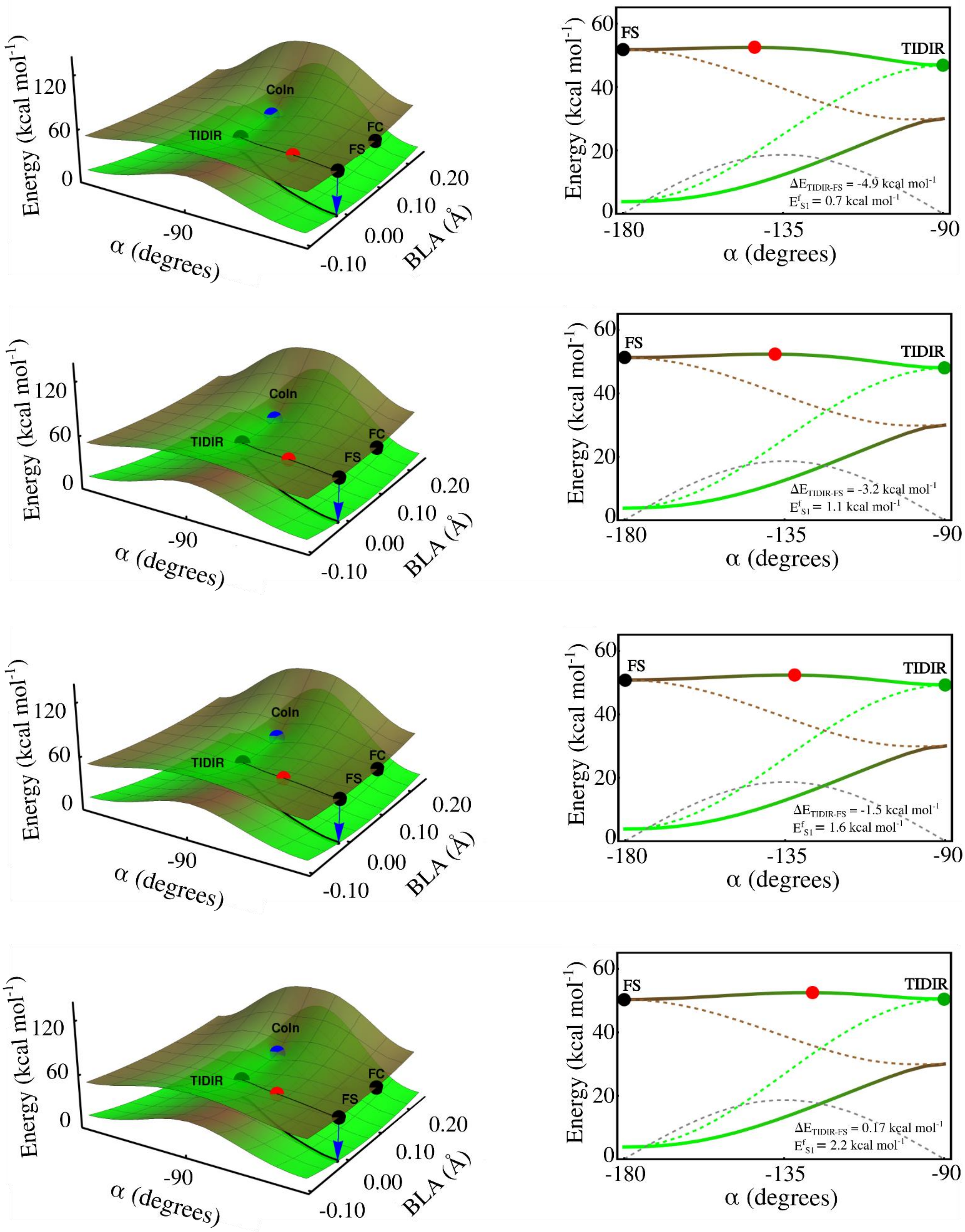
Energy (kcal mol-1)
120
60
0
CoIn
TIDIR
FC
FS
-90
α (degrees)
0.20
0.10
0.00
-0.10
BLA (Å)
Energy (kcal mol-1)
60
40
20
0
FS
TIDIR
ΔETIDIR-FS = -4.9 kcal mol-1
EfS1 = 0.7 kcal mol-1
-180
-135
-90
α (degrees)
ΔETIDIR-FS = -3.2 kcal mol-1
EfS1 = 1.1 kcal mol-1
ΔETIDIR-FS = -1.5 kcal mol-1
EfS1 = 1.6 kcal mol-1
ΔETIDIR-FS = 0.17 kcal mol-1
EfS1 = 2.2 kcal mol-1

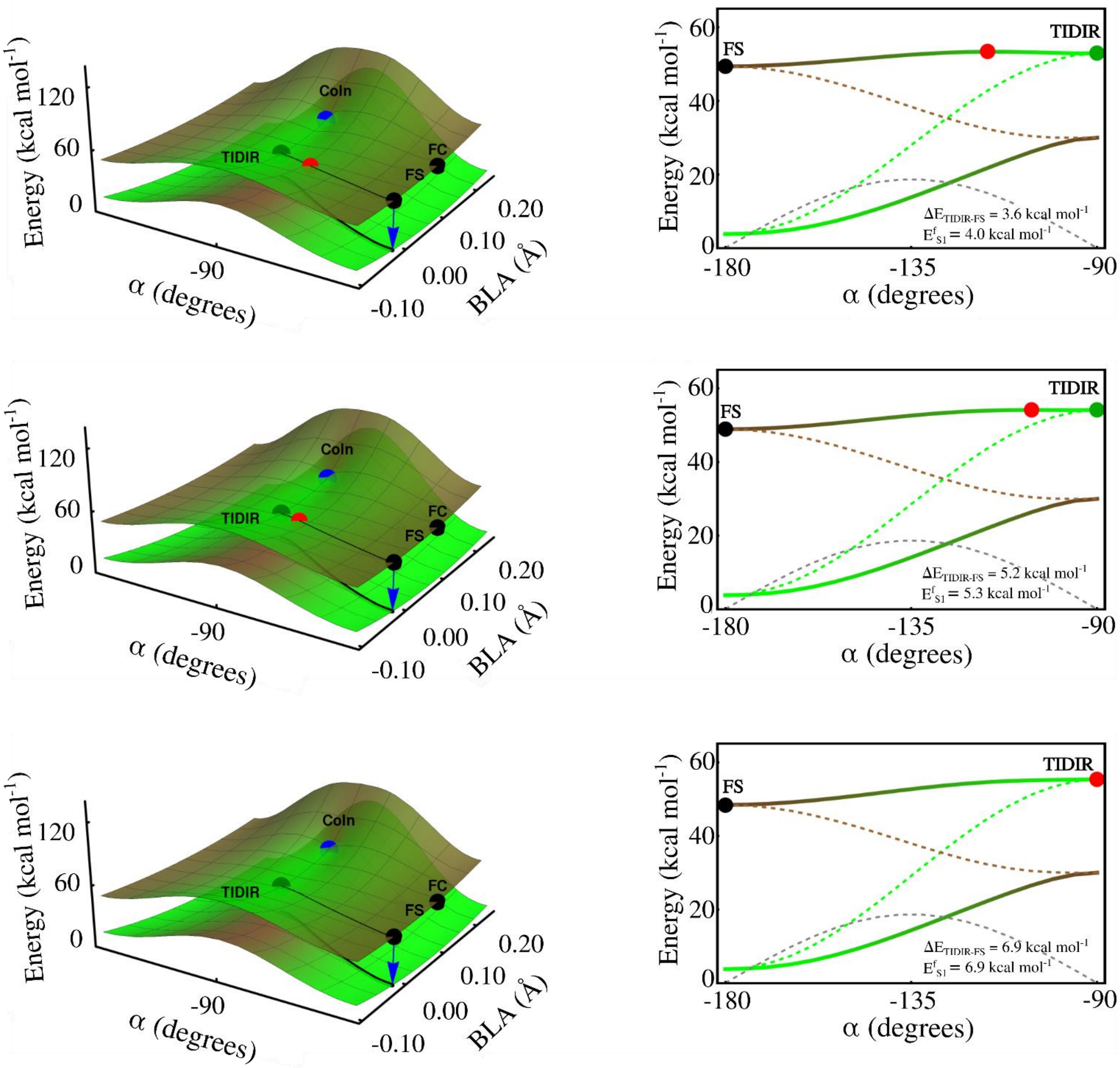

Supplementary Figure 13. **Diabatic representation of the Arch set PESs.** Change in the S_1 and S_0 PESs topography, computed using a 2-state 2-mode model Hamiltonian, as a function of the parameter n. The first row shows the PESs with n=-1, while the last row with n=6. In the first column we plot the PESs along the α and r modes. The color depicts the percentage of diabatic character: brown and green encode the CT and COV/DIR character respectively. Left column: position of the five *critical* S_1 PES points FC, FS in black, TIDIR in green, CoIn in blue and TS_{S1} in red plotted as a function of the parameter n. Right column: diabatic and adiabatic energy profiles along the S_1 MEP connecting FS to TIDIR and comprising TS_{S1}. The energies of adiabatic states correspond to full lines. The energies of diabatic states of equation (15) are represented with dashed lines. The off-diagonal element, Hcp, representing the electronic coupling is shown with a dashed grey line. Here, the diabatic states corresponds to either CT (brown) or COV/DIR (green) electronic characters. The E^f_{S1} and $\Delta E_{TIDIR-FS}$ values for each term of the series are given and, due to the model parametrization, reproduce the trend computed for the Arch series.

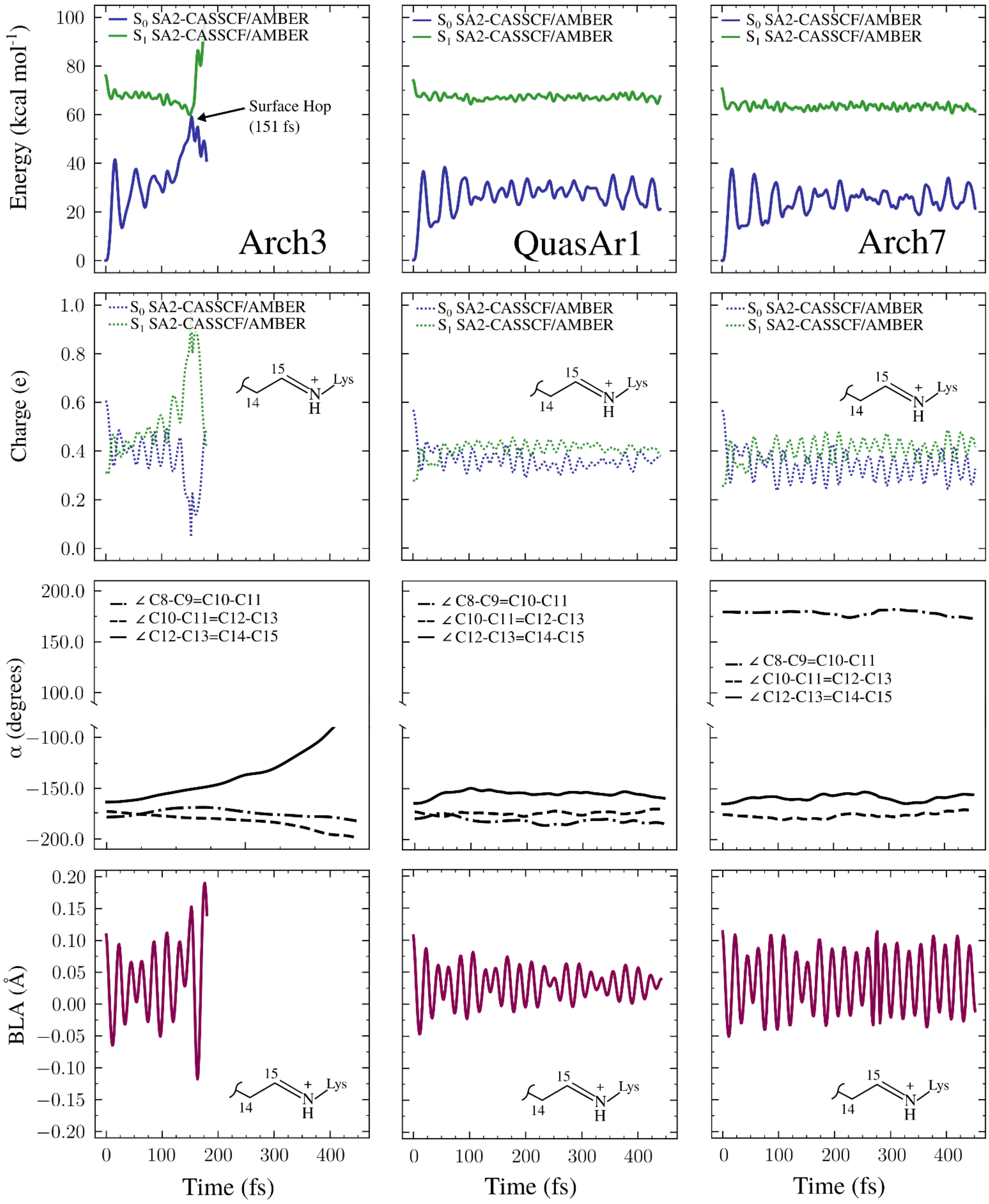

Supplementary Figure 14. FC trajectories of the Arch models. Overview of the results extracted from the FC trajectories propagated on the S_1 PES for Arch3 (left column), QuasAr1 (center column) and Arch7 (right column). The energy profiles (top row) are given at the SA2-CASSCF/AMBER level. The second row displays the evolution of the corresponding fractional Mulliken charge associated to the depicted C14-C15-N moiety for the corresponding wavefunction. The third row shows the time evolution of the main torsional coordinates of the chromophore π-system and include α (the C12-C13-C14-C15 dihedral). A significant deviation from planarity is observed only in correspondence of an isomerization event, as in the case of Arch3 which undergoes all-*trans* -> 13-*cis* isomerization. The bottom row shows the evolution of the BLA associated to the same C14-C15-N moiety.

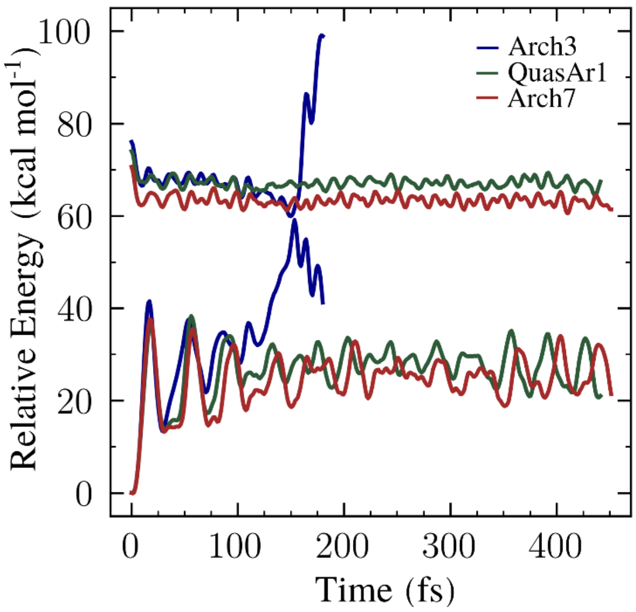

Supplementary Figure 15. Excited state lifetime of the Arch models. SA2-CASSCF/AMBER energy profiles (solid lines) along the FC trajectory for the three representative models Arch3 (blue), QuasAr1 (green) and Arch7 (red). The energies are related to the S_0 energy of the Franck-Condon point. In the profile of the wild-type Arch3, S_0 and S_1 energies become degenerate after ca.151 fs, indicating a surface hopping event has occurred.

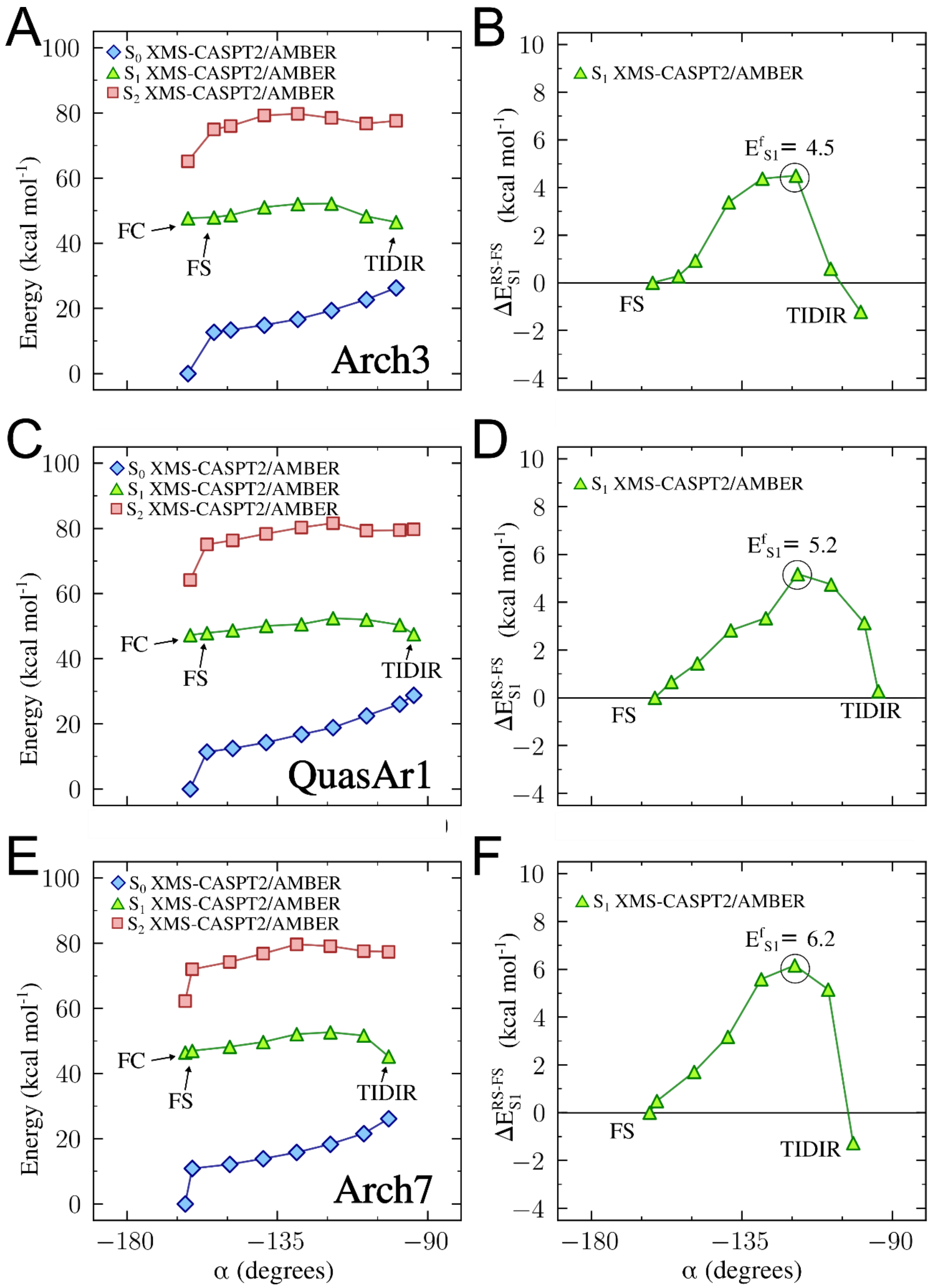

Supplementary Figure 16. *In vacuum* energy profiles along the MEPs calculated at the XMS-CASPT2 level. The geometries were not re-optimized and correspond to those of the corresponding QM/MM reaction paths. The first row shows both the S_0 and S_1 cross sections in Arch3 (A) and the progression of the energy difference between the S_1 energy at value α and S_1 FS energy (B). The highest point along this path corresponds to the quantity E^f_{S1} which is shown in the graph. Same profiles are shown also for QuasAr1 (C, D) and Arch7 (E, F). The energies reported are relative to the S_0 energy of the FC structure.

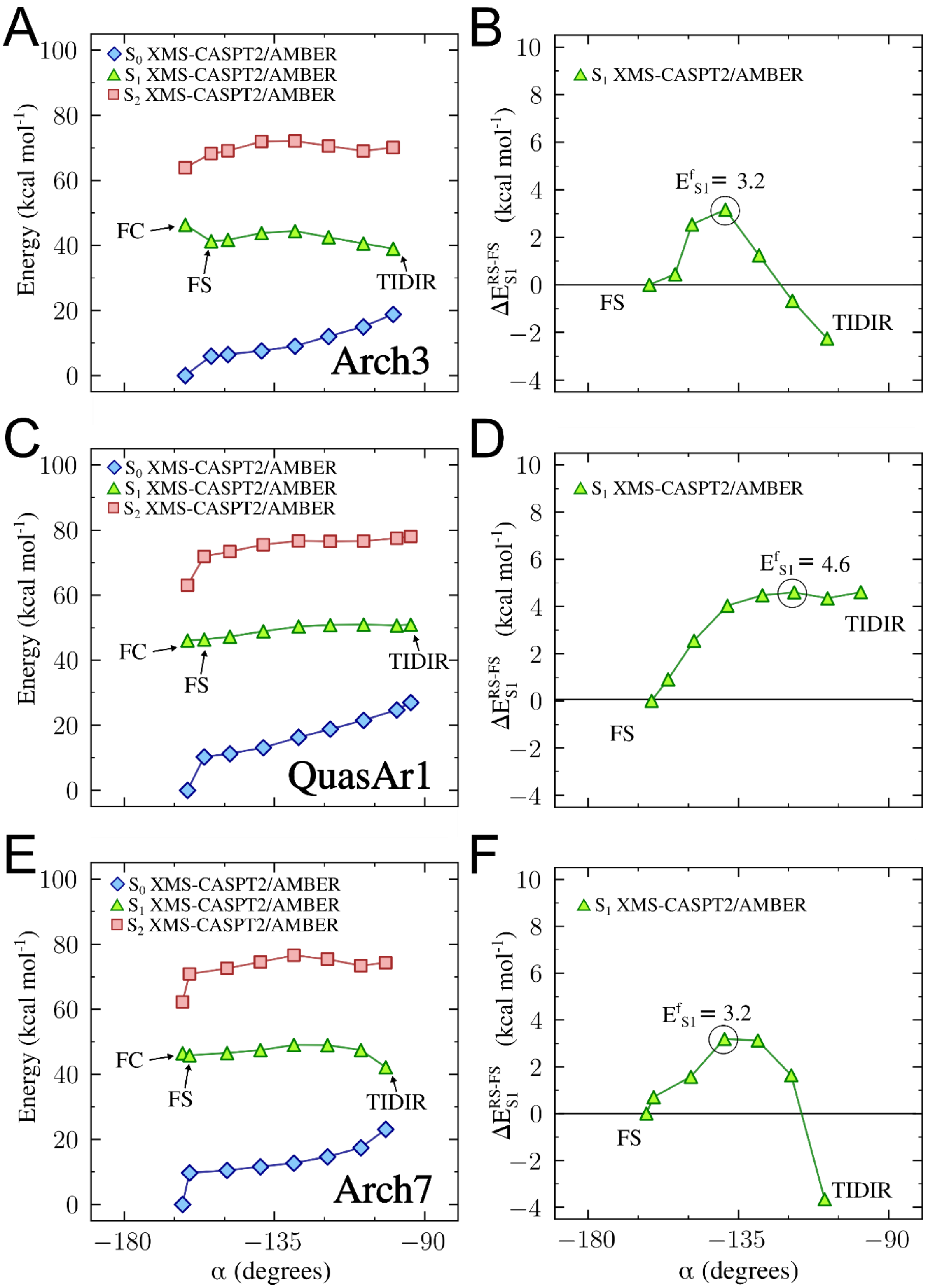

Supplementary Figure 17. Energy profiles along the MEPs calculated neglecting electrostatic interactions calculated at the XMS-CASPT2/AMBER level of theory. The geometries were not re-optimized and correspond to those of the corresponding QM/MM reaction paths. The first row shows both the S_0 and S_1 cross sections in Arch3 (A) and the progression of the energy difference between the S_1 energy at value α and S_1 FS energy (B). The highest point along this path corresponds to the quantity E^f_{S1} which is shown in the graph. Same profiles are shown also for QuasAr1 (C, D) and Arch7 (E, F). The energies reported are relative to the S_0 energy of the FC structure.

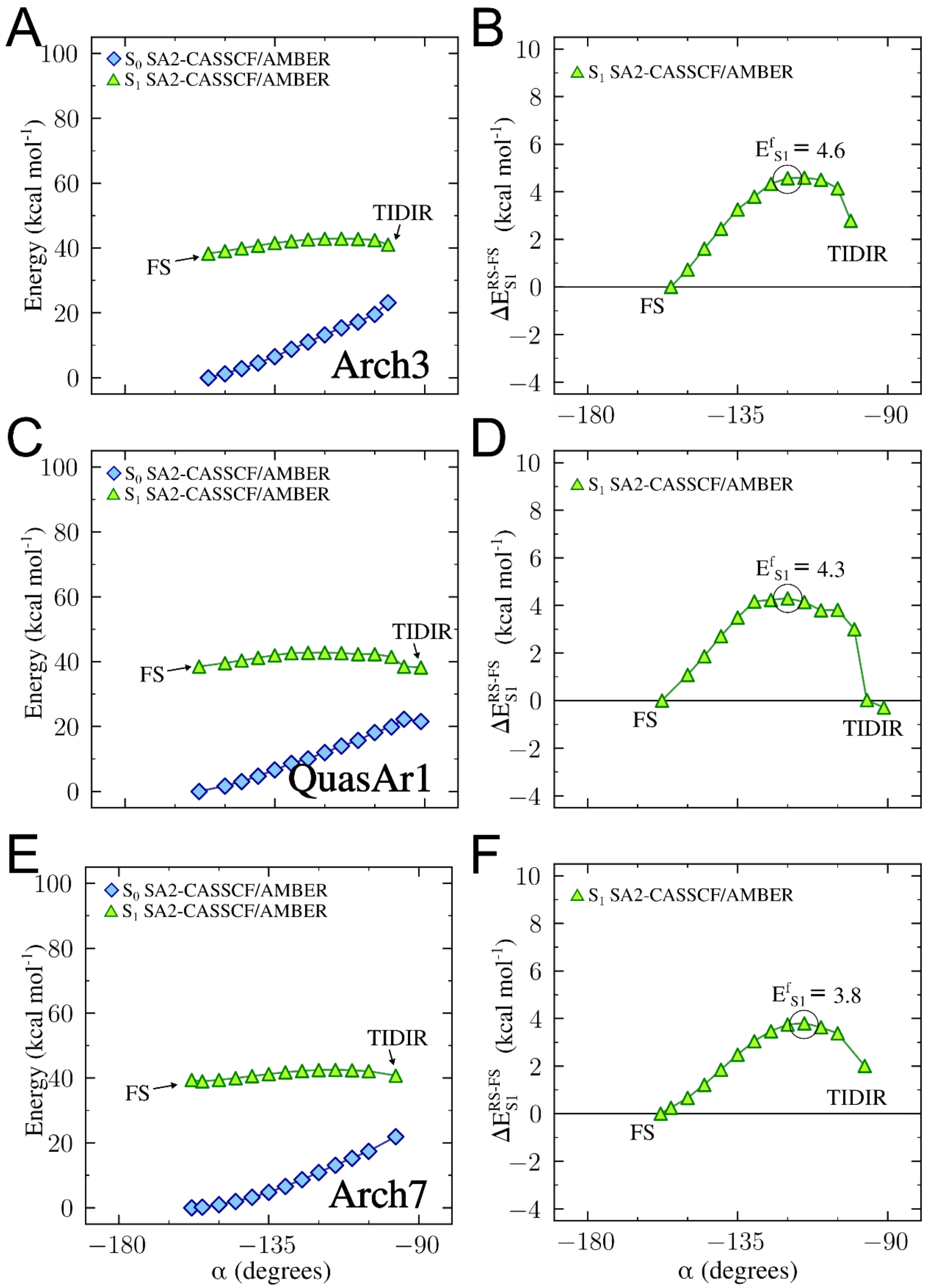

Supplementary Figure 18. Energy profiles along the MEP calculated neglecting electrostatic interactions for Arch3, QuasAr1 and Arch7, calculated at the SA2-CASSCF/AMBER level of theory. The first row shows both the S_0 and S_1 cross sections in Arch3 (A) and the progression of the energy difference between the S_1 energy at value α and S_1 FS energy (B). The highest point along this path corresponds to the quantity E^f_{S1} which is shown in the graph. Same profiles are shown also for QuasAr1 (C, D) and Arch7 (E, F). Geometrical relaxation of the chromophore (QM) and of the cavity amino acids side chains (MM) was allowed, with α constrained at the value used in the original reaction path (i.e., the QMMM path calculated at the same level of theory). The energies reported are relative to the S_0 energy of the FS structure.

Supplementary References

1. Berman, H. M. *et al.* The protein data bank. *Nucleic Acids Res* **28**, 235–242 (2000).
2. Kouyama, T. *et al.* Structure of archaerhodopsin-2 at 1.8 Å resolution. *Acta Crystallographica Section D: Biological Crystallography* **70**, 2692–2701 (2014).
3. Bada Juarez, J. F. *et al.* Structures of the archaerhodopsin-3 transporter reveal that disordering of internal water networks underpins receptor sensitization. *Nat Commun* **12**, 1–10 (2021).
4. McIsaac, R. S. *et al.* Directed evolution of a far-red fluorescent rhodopsin. *Proceedings of the National Academy of Sciences* **111**, 13034–13039 (2014).
5. Piatkevich, K. D. *et al.* A robotic multidimensional directed evolution approach applied to fluorescent voltage reporters. *Nat Chem Biol* **14**, 352–360 (2018).
6. Hochbaum, D. R. *et al.* All-optical electrophysiology in mammalian neurons using engineered microbial rhodopsins. *Nat Methods* **11**, 825–833 (2014).
7. Eswar, N. *et al.* Comparative protein structure modeling using Modeller. *Curr Protoc Bioinformatics* **15**, 5–6 (2006).
8. Shen, M. & Sali, A. Statistical potential for assessment and prediction of protein structures. *Protein science* **15**, 2507–2524 (2006).
9. Melaccio, F. *et al.* Toward automatic rhodopsin modeling as a tool for high-throughput computational photobiology. *Journal of Chemical Theory and Computation* **12**, 6020–6034 (2016).
10. Pedraza-González, L., de Vico, L., del Carmen Marín, M., Fanelli, F. & Olivucci, M. a-ARM: automatic rhodopsin modeling with chromophore cavity generation, ionization state selection, and external counterion placement. *J Chem Theory Comput* **15**, 3134–3152 (2019).
11. del Carmen Marín, M. *et al.* Fluorescence enhancement of a microbial rhodopsin via electronic reprogramming. *J Am Chem Soc* **141**, 262–271 (2018).
12. Ernst, O. P. *et al.* Microbial and animal rhodopsins: structures, functions, and molecular mechanisms. *Chem Rev* **114**, 126–163 (2014).
13. Strickler, S. J. & Berg, R. A. Relationship between absorption intensity and fluorescence lifetime of molecules. *J Chem Phys* **37**, 814–822 (1962).
14. Olsen, J. The CASSCF method: A perspective and commentary. *International Journal of Quantum Chemistry* **111**, 3267–3272 (2011).
15. Martin, M. E., Negri, F. & Olivucci, M. Origin, nature, and fate of the fluorescent state of the green fluorescent protein chromophore at the CASPT2//CASSCF resolution. *J Am Chem Soc* **126**, 5452–5464 (2004).
16. Ferré, N. & Olivucci, M. Probing the rhodopsin cavity with reduced retinal models at the CASPT2//CASSCF/AMBER level of theory. *J Am Chem Soc* **125**, 6868–6869 (2003).
17. Cattaneo, P. & Persico, M. An ab initio study of the photochemistry of azobenzene. *Physical Chemistry Chemical Physics* **1**, 4739–4743 (1999).
18. Shiozaki, T., Győrffy, W., Celani, P. & Werner, H.-J. Communication: Extended multi-state complete active space second-order perturbation theory: Energy and nuclear gradients. *The Journal of Chemical Physics* **135**, 81106 (2011).
19. Granovsky, A. A. Extended multi-configuration quasi-degenerate perturbation theory: The new approach to multi-state multi-reference perturbation theory. *J Chem Phys* **134**, 214113 (2011).
20. Manathunga, M., Yang, X. & Olivucci, M. Electronic state mixing controls the photoreactivity of a rhodopsin with all-trans chromophore analogues. *J Phys Chem Lett* **9**, 6350–6355 (2018).

21. Page, C. S. & Olivucci, M. Ground and excited state CASPT2 geometry optimizations of small organic molecules. *J Comput Chem* **24**, 298–309 (2003).
22. Valsson, O. & Filippi, C. Photoisomerization of model retinal chromophores: insight from quantum monte carlo and multiconfigurational perturbation theory. *Journal of Chemical Theory and Computation* **6**, 1275–1292 (2010).
23. Gozem, S. *et al.* Shape of multireference, equation-of-motion coupled-cluster, and density functional theory potential energy surfaces at a conical intersection. *J Chem Theory Comput* **10**, 3074–3084 (2014).
24. Filatov, M. & Shaik, S. A spin-restricted ensemble-referenced Kohn–Sham method and its application to diradicaloid situations. *Chem Phys Lett* **304**, 429–437 (1999).
25. Huix-Rotllant, M. *et al.* Assessment of density functional theory for describing the correlation effects on the ground and excited state potential energy surfaces of a retinal chromophore model. *J Chem Theory Comput* **9**, 3917–3932 (2013).
26. Gozem, S., Luk, H. L., Schapiro, I. & Olivucci, M. Theory and simulation of the ultrafast double-bond isomerization of biological chromophores. *Chem Rev* **117**, 13502–13565 (2017).
27. Liang, R., Liu, F. & Mart\'\inez, T. J. Nonadiabatic photodynamics of retinal protonated Schiff base in Channelrhodopsin 2. *J Phys Chem Lett* **10**, 2862–2868 (2019).
28. Yu, J. K., Liang, R., Liu, F. & Mart\'\inez, T. J. First-principles characterization of the elusive i fluorescent state and the structural evolution of retinal protonated schiff base in bacteriorhodopsin. *J Am Chem Soc* **141**, 18193–18203 (2019).
29. Liang, R., Yu, J. K., Meisner, J., Liu, F. & Martinez, T. J. Electrostatic Control of Photoisomerization in Channelrhodopsin 2. *J Am Chem Soc* **143**, 5425–5437 (2021).
30. Lu, T. & Chen, F. Multiwfn: a multifunctional wavefunction analyzer. *J Comput Chem* **33**, 580–592 (2012).
31. Fdez. Galván, I. *et al.* OpenMolcas: From Source Code to Insight. *Journal of Chemical Theory and Computation* **15**, 5925–5964 (2019).
32. A. A. Granovsky, Firefly version 8.2, www http://classic.chem.msu.su/gran/firefly/index.html.
33. Marsili, E., Farag, M. H., Yang, X., De Vico, L. & Olivucci, M. Two-State, Three-Mode Parametrization of the Force Field of a Retinal Chromophore Model. *Journal of Physical Chemistry A* **123**, 1710–1719 (2019).
34. Gozem, S., Ling Luk, H., Schapiro, I. & Olivucci, M. Theory and Simulation of the Ultrafast Double-Bond Isomerization of Biological Chromophores. *Chemical Reviews* **117**, 13502–13565 (2017).
35. Aquilante, F. *et al.* Molcas 8: New capabilities for multiconfigurational quantum chemical calculations across the periodic table. *Journal of Computational Chemistry* **37**, 506–541 (2016).
36. Virtanen, P. *et al.* SciPy 1.0: fundamental algorithms for scientific computing in Python. *Nat Methods* **17**, 261–272 (2020).
37. Byrd, R. H., Schnabel, R. B. & Shultz, G. A. A trust region algorithm for nonlinearly constrained optimization. *SIAM Journal on Numerical Analysis* **24**, 1152–1170 (1987).
38. Enami, N. *et al.* Crystal structures of archaerhodopsin-1 and-2: Common structural motif in archaeal light-driven proton pumps. *J Mol Biol* **358**, 675–685 (2006).
39. Penzkofer, A., Silapetere, A. & Hegemann, P. Photocycle dynamics of the Archaerhodopsin 3 based fluorescent voltage sensor QuasAr1. *Int J Mol Sci* **21**, 160 (2019).
40. saint Clair, E. C. *et al.* Near-IR resonance Raman spectroscopy of archaerhodopsin 3: effects of transmembrane potential. *The Journal of Physical Chemistry B* **116**, 14592–14601 (2012).
