# Supplementary material for: On the fluorescence enhancement of arch neuronal optogenetic reporters
Source: Nat Commun. 2022 Oct 28;13:6432. doi: 10.1038/s41467-022-33993-4 (PMC9616920; doi:10.1038/s41467-022-33993-4)
Supplement: Supplementary file 3 — Description of Additional Supplementary Files [file 41467_2022_33993_MOESM3_ESM.pdf]

## Description of Additional Supplementary Files

File Name: Supplementary Data 1
Description:

Cartesian coordinates (in Tinker format) of the FC, FS, CoIn and TIDIR geometries of the QM/MM models calculated at the SA2-CASSCF(12,12)/6-31G*/AMBER level of theory. The models correspond to the Arch set (Arch2, Arch3, QuasAr1, Archon2, QuasAr2, Arch7, Arch5).

File Name: Supplementary Data 2
Description:

Cartesian coordinates (in Tinker format) of the FC, FS and TIDIR geometries of the QM/MM models calculated at the XMS-CASPT2/SA3-CASSCF(12,12)/ANO-L-vDZP/AMBER level of theory. The models correspond to the Arch set (Arch2, Arch3, QuasAr1, Archon2, QuasAr2, Arch7, Arch5).
